# mir15a/mir16‐1 cluster and its novel targeting molecules negatively regulate cardiac hypertrophy

**DOI:** 10.1002/ctm2.242

**Published:** 2020-12-15

**Authors:** Hongchang Guo, Ke Ma, Wenjing Hao, Yao Jiao, Ping Li, Jing Chen, Chen Xu, Fu‐jian Xu, Wayne Bond Lau, Jie Du, Xin‐liang Ma, Yulin Li

**Affiliations:** ^1^ Beijing Anzhen Hospital of Capital Medical University and Beijing Institute of Heart Lung and Blood Vessel Diseases Beijing China; ^2^ State Key Laboratory of Chemical Resource Engineering, and Beijing Laboratory of Biomedical Materials Beijing University of Chemical Technology Beijing China; ^3^ Department of Emergency Medicine Thomas Jefferson University Philadelphia Pennsylvania

**Keywords:** biomarkers, cardiac hypertrophy, heart failure, miRNAs, therapeutic target

## Abstract

In response to pathological stimuli, the heart develops ventricular hypertrophy that progressively decompensates and leads to heart failure. miRNAs are increasingly recognized as pathogenic factors, clinically relevant biomarkers, and potential therapeutic targets. We identified that mir15a/mir16‐1 cluster was negatively correlated with hypertrophic severity in patients with hypertrophic cardiomyopathy. The mir15a/mir16‐1 expression was enriched in cardiomyocytes (CMs), decreased in hypertrophic human hearts, and decreased in mouse hearts after transverse aortic constriction (TAC). CM‐specific mir15a/mir16‐1 knockout promoted cardiac hypertrophy and dysfunction after TAC. CCAAT/enhancer binding protein (C/EBP)β was responsible for the downregulation of mir15a/mir16‐1 cluster transcription. Mechanistically, mir15a/mir16‐1 cluster attenuated the insulin/IGF1 signal transduction cascade by inhibiting multiple targets, including INSR, IGF‐1R, AKT3, and serum/glucocorticoid regulated kinase 1 (SGK1). Pro‐hypertrophic response induced by mir15a/mir16‐1 inhibition was abolished by knockdown of insulin receptor (INSR), insulin like growth factor 1 receptor (IGF1R), AKT3, or SGK1. In vivo systemic delivery of mir15a/mir16‐1 by nanoparticles inhibited the hypertrophic phenotype induced by TAC. Importantly, decreased serum mir15a/mir16‐1 levels predicted the occurrence of left ventricular hypertrophy in a cohort of patients with hypertension. Therefore, mir15a/mir16‐1 cluster is a promising therapeutic target and biomarker for cardiac hypertrophy.

## Graphical abstract

mir15a/mir16‐1 cluster exerts protective effects against the progression of the cardiac hypertrophy and dysfunction (left). During hypertrophic stress, increased C/EBPβ downregulates the mir15a/mir16‐1 cluster, resulting in up‐regulation of multiple target proteins (INSR, IGF1R, AKT3, SGK1) in cardiomyocytes, causing increased activation of insulin/IGF1 signaling, ultimately causing cardiac hypertrophy and dysfunction. The CHO‐PEGA delivery system replenishes mir15a/mir16‐1 in the heart, attenuating cardiac hypertrophy and heat failure (right). Meanwhile, reduced circulating mir15a/mir16‐1 levels are associated with the hypertrophic degree and cardiac hypertrophy risk in patients (bottom).

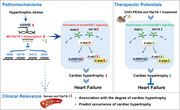

**Abbreviations**
AKT: serine/threonine kinase
BNP: B‐type natriuretic peptide
C/EBP: CCAAT/enhancer binding protein
CKO: cardiomyocyte‐specific knockout
ERK1: mitogen‐activated protein kinase 1
IGF1R: insulin like growth factor 1 receptor
INSR: insulin receptor
mTOR: mammalian target of rapamycin
mTORC1: mammalian target of rapamycin complex 1
qRT‐PCR: quantitative real‐time PCR
SGK1: serum/glucocorticoid regulated kinase 1
SMAD3: SMAD Family Member 3
STAT3: signal transducer and activator of transcription 3
TGF: transforming growth factor
WT: wild type

## 1 INTRODUCTION

Cardiac hypertrophy and consequent heart failure are significant causes of mortality worldwide. In response to pathological stress such as pressure/volume overload, neurohumoral stimuli, and inherited mutations, the heart mounts a hypertrophic response characterized by enlargement of cardiomyocytes (CMs).^1^, ^2^ Although initial cardiac hypertrophy can be viewed as a compensatory response to increase cardiac output against excessive afterload, persistent hypertrophy is mainly maladaptive, ultimately impairing contractility and driving heart failure progression.^3^ Multiple preclinical studies have reported that blockade of hypertrophic cardiac growth improves heart function.^4^ The list of identified molecular factors that contribute to hypertrophy is growing. However, translating such molecular targets to new therapies against cardiac hypertrophy is difficult, largely due to fundamental differences between human and animal models. Thus, seeking the key regulators of pathological hypertrophy most relevant to patients is important to develop new efficacious treatment strategies.

miRNA may provide novel diagnostic and therapeutic tools for cardiovascular disease.^5^, ^6^ It is interesting that several of the most abundant miRNAs in the heart belong to families, including let‐7‐, mir15‐, mir29‐, and mir30‐families,^7^ of which let‐7‐, mir30‐, and mir 29‐families have been fully investigated and understood in pathological ventricular remodeling.^8^, ^9^, ^10^ The mir15 family has been shown to play very important roles in heart regeneration and development,^11^ but relatively few in pathological stress. In addition, pathological stress influences the production and release of miRNAs into circulation from the heart and/or other organs, altering the circulating miRNA pool. The circulating levels of these cardiac‐enriched miRNAs, such as mir29a, correlated with hypertrophy and fibrosis in patients with hypertrophic cardiomyopathy (HCM).^12^ Thus, whether there are more functionally important miRNAs that serve as hypertrophic biomarkers and carry a pathogenic role in cardiac hypertrophy remains to be determined.

**Highlights**

- Cardiomyocyte (CM)‐specific mir15a/mir16‐1 protects against cardiac hypertrophy and sequelae (e.g. heart failure) induced by pressure overload, even aging.
- C/EBPβ acts as the upstream molecule causing transcriptional downregulation of mir15a/mir16‐1 cluster in the CMs.
- Prospective study revealed that decreased mir15a/mir16‐1 levels are a predictive biomarker for the occurrence of left ventricular hypertrophy (LVH).

In this study, we screened hypertrophic‐associated miRNAs. We demonstrate the mir15a/mir16‐1 cluster is negatively correlated with the degree of cardiac hypertrophy in patients with HCM. Additionally, CCAAT/enhancer binding protein (C/EBP)β is responsible for its downregulation in CMs by directly binding to its promoters. We demonstrate CM‐specific mir15a/mir16‐1 exhibits a protective role against the development of cardiac hypertrophy and dysfunction by suppressing insulin‐IGF1 signaling. Finally, our clinical data suggest that serum mir15a/mir16‐1 concentration may aid in determining the likelihood of left ventricular hypertrophy (LVH) development.

## 2 METHODS

### 2.1 Study design

The overall objective of this study was to identify miRNAs as regulators and biomarkers of pathological cardiac hypertrophy, and to develop a therapeutic approach using nanoparticle‐carrying miRNA delivery. We performed studies in patients with cardiac hypertrophy, in mouse models of pressure overload induced by transverse aortic constriction (TAC), and in cultured CMs. The following experimental studies were designed: (a) To identify the miRNAs associated with hypertrophic development, we performed an unbiased circulating miRNA sequencing in patients with HCM (with and without obstruction) and healthy control (HC), and that was subsequently independently validated using quantitative real‐time PCR (qRT‐PCR). The relationship between serum miRNA levels and hypertrophic severity was evaluated. These experiments established circulating mir15a/mir16‐1 cluster associated with human cardiac hypertrophy. (b) The expression of mir15a/mir16‐1 cluster was determined in both human and mouse hypertrophic hearts by qRT‐PCR, and its cellular localization was assessed in mouse by in situ hybridization (ISH). In addition, we investigated the transcriptional mechanisms of mir15a/16‐1 downregulation under hypertrophic stress by in‐silico prediction analysis, luciferase reporter assays, and ChIP‐PCR. (c) To explore the functional consequences of mir15a/mir16‐1 in cardiac hypertrophy, we generated tamoxifen‐inducible, CM‐specific miRNA‐knockout mice, and applied a mouse model of pressure overload induced by TAC. Cardiac hypertrophy, fibrosis, and heart function were evaluated by histopathological analysis and echocardiogram test. (d) A series of bioinformatics and experimental steps were performed to identify direct and novel targets of mir15a/mir16‐1 cluster, which were responsible for the effect on cardiac hypertrophy. (e) To determine the potential therapeutic role, mir15a/mir16‐1 cluster was delivered by nanoparticles into the TAC animal model. We examined the in vitro and in vivo effects of mir15a/mir16‐1 replenishment in cardiac hypertrophy. To further characterize the global impact of mir15a/mir16‐1 treatment, we assessed the cardiac protein profile by proteomics analysis. (f) The dysregulation of mir15a/mir16‐1 in serum and the capability of circulating mir15a/mir16‐1 to predict the occurrence of LVH were assessed by qRT‐PCR and COX regression analysis in a cohort of patients with hypertension.

### 2.2 Studies in patients

Serum levels of miRNAs were ascertained from HC and patients with HCM or hypertension recruited from Beijing Anzhen Hospital. LV tissues were obtained from normal heart donors or patients diagnosed with OHCM undergoing septal myotomy surgery. The Institutional Review Board of the Institute of Biophysics and Beijing Anzhen Hospital approved all human studies involving cardiac tissue and blood samples. Written informed consent was obtained from all participants. A detailed description of the study population and human sample collection are reported in the supporting material online.

### 2.3 Studies in mouse model

All animal studies were performed in accordance with protocols approved by the Animal Subjects Committee of Beijing Anzhen Hospital. Mice carrying floxed alleles of mir15a/mir16‐1 (Mirc30tm1.1Rdf/J mice)^13^, ^14^ were gifted by Dr. Yiwei Chu (Fudan University). The mice carrying floxed alleles of mir15a/mir16‐1 were backcrossed to a C57BL/6 background at least ten times. The generation of Myh6‐cre/Esr1^+/−^ mice has been reported previously.^15^ The tamoxifen‐inducible, CM‐specific mir15a/mir16‐1 knockout mice were generated by intercrossing the Myh6‐cre/Esr1^+/−^ mice with Mirc30tm1.1Rdf/J mice. Left pressure overload was induced in cardiomyocyte‐specific knockout (CKO) and wild type (WT) mice by partial ligation of the aorta between the innominate and left common carotid arteries, causing constant and permanent constriction. Animal details, serial echocardiography, morphological, and immunohistochemical experiments are reported in the supporting material online.

### 2.4 Statistics

Values are expressed as the mean ± SD. The Shapiro‐Wilk test was used to evaluate the normality of data distribution. Differences between two groups were analyzed by the Student's two‐sided *t*‐test (for normally distributed continuous variables, with log transformation as required), the Mann‐Whitney *U* test (for non‐normally distributed continuous variables), and chi‐square test (for categorical data). One‐way ANOVA combined with post‐hoc Tukey's test was used for comparisons between more than two groups. Differences between groups over time were evaluated using repeated measures analysis of variance. Detailed statistical analytic methods about clinical study are reported in the supporting material online.

### 2.5 Other protocols

Detailed methodology for all protocols used in this study (including miRNA‐sequencing, chromatin immunoprecipitation assays, luciferase reporter assays, in vitro and in vivo delivery of mir15a/mir16‐1, transcriptomic and proteomics analysis, and others) is provided in the supporting material online.

## 3 RESULTS

### 3.1 Clinical evidence demonstrating the negative association between mir15a/mir16‐1 cluster and cardiac hypertrophy

**FIGURE 1:**
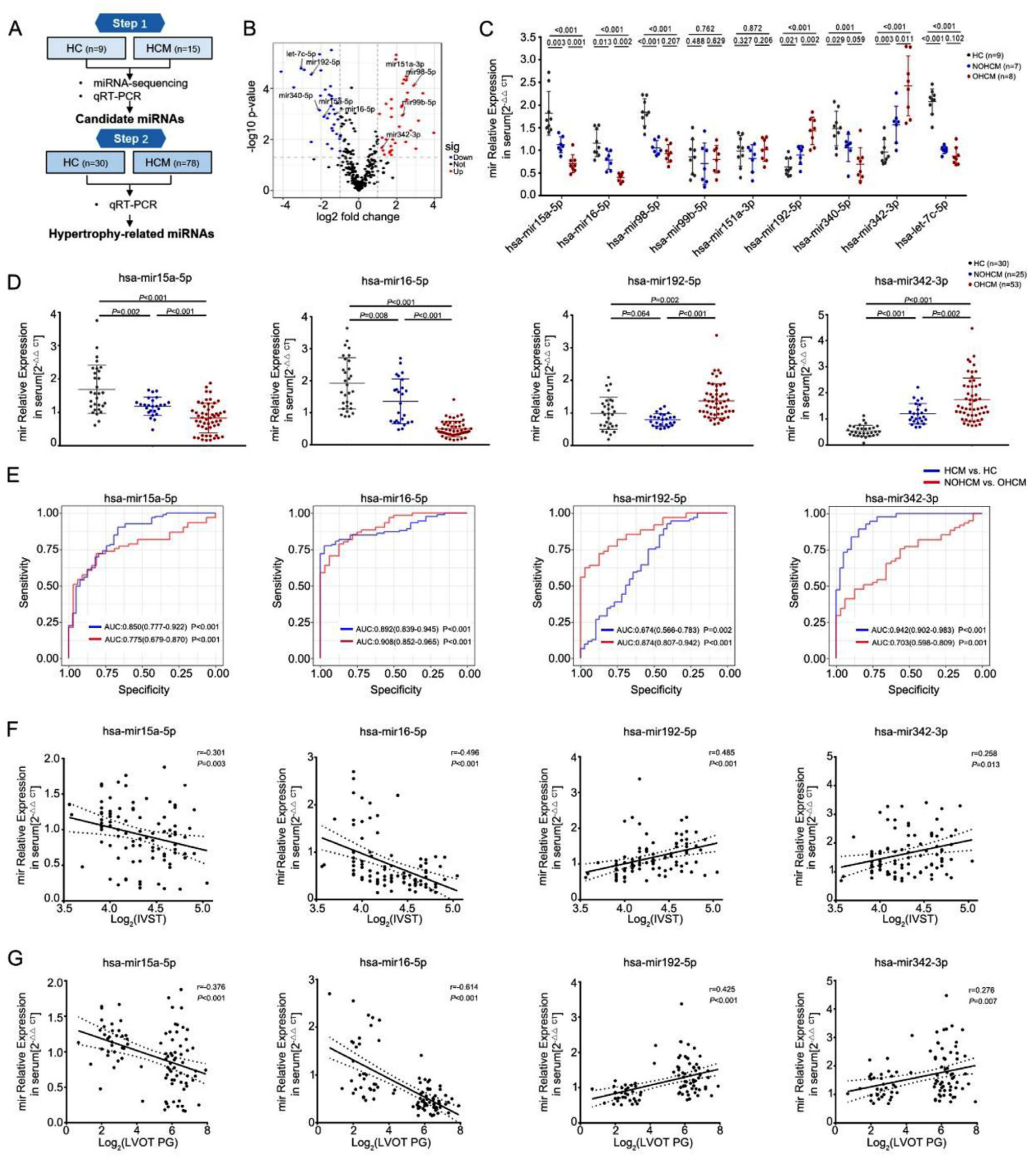
**Identification of hypertrophy‐related miRNAs in patients with HCM. A,** Schematic description of the workflow illustrating the two‐stage‐approach involving independent samples for discovery and validation. **B,** Volcano plot revealing miRNA‐sequencing results comparing HCM (n = 15) versus HCs (n = 9). Individual miRNAs are displayed by the FDR‐adjusted *P*‐value and the corresponding fold change. **C,** qRT‐PCR espression analysis of nine candidate miRNAs in discovery samples (HCs = 9, NOHCM = 7, OHCM = 8). **D,** qRT‐PCR expression analysis of four miRNAs (mir15a‐5p, mir16‐5p, mir192‐5p, and mir342‐3p) in an independent validation sample (HCs = 30, NOHCM = 25, OHCM = 53). **E,** Area under the receiver operating curve of 4 miRNAs (values given on the graphs) discriminating HCM versus HC, NOHCM versus OHCM. **F and G,** Correlation analysis of the expression of single miRNA and the interventricular septum thickness (IVST) (**F**) and the left ventricular outflow tract (LVOT) pressure gradient (PG) (**G**) in patients with HCM (n = 93). Statistical significance was determined by 1‐way ANOVA Tukey's post‐hoc test (C and D) by Spearman's correlation test (F and G) Abbreviations: HC, healthy control; HCM, hypertrophic cardiomyopathy; NOHCM, non‐obstructive hypertrophic cardiomyopathy; OHCM, obstructive hypertrophic cardiomyopathy.

We designed a two‐stage case‐control study to investigate hypertrophy‐related miRNAs (Figure 1A). The demographic and clinical characteristics of the discovery and validation samples are shown (Tables S1‐S2). First, we performed pilot miRNA‐sequencing using serum from HC (n = 9) and patients with HCM (eight obstructive HCM [OHCM] patients and seven non‐obstructive HCM [NOHCM] patients). Nine candidate miRNAs between HC and HCM were selected based on the following three criteria: (a) a false discovery rate‐adjusted *P*‐value <.05; (b) fold change ≥2.0; (c) miRNA expression abundance >100 reads per million (Figure 1B). Second, nine miRNAs were tested by qRT‐PCR in these 24 samples. There are significant differences in four miRNAs (mir16‐5p, mir15a‐5p, mir192‐5p, and mir342‐3p) between any two of three groups (including HC vs NOHCM, HC vs OHCM, NOHCM vs OHCM) (Figure 1C). Third, we examined four candidate miRNAs expression in independent validation samples (53 OHCM patients, 25 NOHCM patients, and 30 HC) by qRT‐PCR. Four miRNAs exhibited consistent directionality with the first qRT‐PCR results in the discovery set (Figure 1D). Using the receiver‐operating characteristic curve (ROC) and Spearman's correlation analyses, we calculated the discrimination ability and correlation with hypertrophic severity of four miRNAs. Candidate miRNAs would be selected based on the two criteria: (a) ROC analysis: AUC > 0.7, *P* < .05 between two comparisons (HC vs HCM, NOHCM vs OHCM; (b) correlation analysis: r > 0.3, *P* < .05. The area under the ROC (AUC) values of four miRNAs ranged from 0.703 to 0.908 between NOHCM and OHCM, with mir16‐5p exhibiting the greatest discriminatory power (AUC: 0.908 [95% CI 0.852‐0.965, where CI is confidence interval], *P* < .001) (Figure 1E). The interventricular septum thickness (IVST) and the left ventricular outflow tract (LVOT) pressure gradient (PG) serve as quantitative markers of hypertrophic degree. Consistent and significant negative correlations with IVST and LVOT PG were demonstrated for mir15a‐5p and mir16‐5p, but a positive correlation was demonstrated for mir192‐5p and mir342‐3p (Figure 1F‐G). mir16‐5p exhibited the strongest correlation with hypertrophic degree, as evidenced by IVST (r = −0.496, *P* < .001) and LVOT PG (r = −0.614, *P* < .001). Finally, mir15a and mir16‐5p were choosen as a good discrimination ability and correlation. mir15a‐5p and mir16‐5p were transcribed as a cluster (mir15a/mir16‐1), residing in the 13q14 chromosomal region. Both miRNAs displayed a common seed sequence and shared 80% complementarity.^16^ To explore circulating mir15a‐5p and mir16‐5p location, we tested their expression in the exosomes isolated from plasma from HCM patients and HC. The levels of mir15a‐5p and mir16‐5p were lower in the exosomes than in plasma, but levels in plasma‐derived exosomes did not differ between HCM patients and HC (Figure S1). Collectively, decreased levels of the serum mir15a/mir16‐1 cluster correlated strongly with hypertrophic severity in HCM patients, suggesting that mir15a/mir16‐1 cluster alteration was likely attributable to cardiac hypertrophy.

Having demonstrated that circulating mir15a/mir16‐1 is downregulated in HCM patients, we next determined the organ from which circulating mir15a/mir16‐1 originated. We evaluated mir15a/mir16‐1 expression in cardiac tissue from both humans and mice after TAC. Expression of mir15a and mir16‐1 was significantly downregulated in OHCM patient hearts compared to that in normal control (Figure S2A). In animals subjected to TAC, cardiac expression of both mir15a and mir16‐1 rapidly declined 2 weeks after TAC, remaining significantly downregulated thereafter (Figure S2B). Finally, to ascertain the cellular origin of cardiac mir15a/mir16‐1, we determined the expression of mir15a/mir16‐1 in isolated neonatal CMs, cardiac fibroblasts, endothelial cells, and vascular smooth muscle cells. Using qRT‐PCR ISH, we determined CMs significantly increased mir15a and mir16‐1 expression (Figure S3A‐C).

### 3.2 Exploring the causative role of mir15a/mir16‐1 in hypertrophy development

**FIGURE 2:**
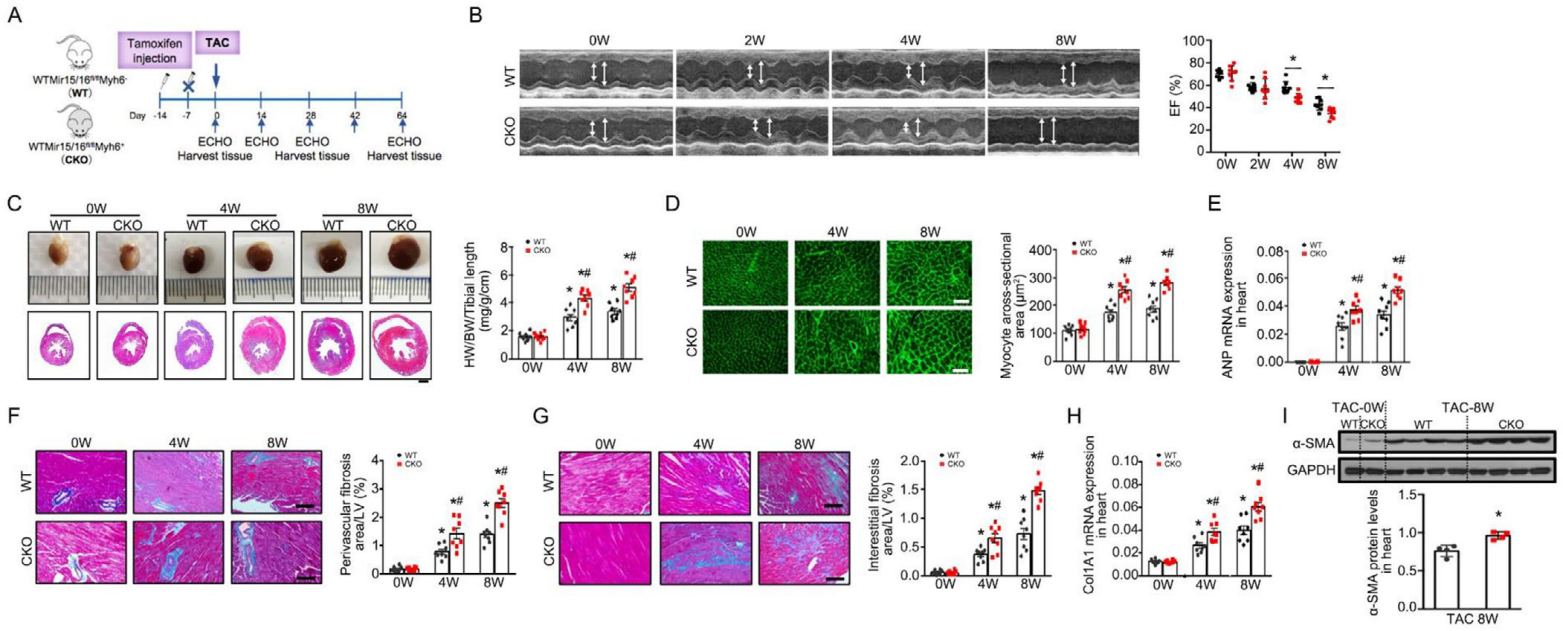
**Cardiomyocyte‐specific mir15a/mir16‐1 knockout aggravates cardiac hypertrophy and dysfunction after TAC. A,** Cardiomyocyte‐specific mir15a/mir16‐1 knockout (CKO) and wild‐type (WT) control mice were subjected to tamoxifen, followed by TAC operation for 8 weeks. **B,** M‐mode echocardiographic imaging of the heart before (0) and after (2, 4, and 8 weeks) TAC. Analysis of and ejection fraction (EF)% of hearts of WT and CKO mice subjected to TAC (n = 8 per group). *P < .05 versus WT mice. **C,** Whole‐mount representation of heart (Upper), representative image of heart sections stained with Masson trichrome (bottom), heart weight/body weight/tibia length ratios in WT and CKO mice before and after (4 and 8 weeks) TAC (n = 8 per group). *P < .05 versus mice before TAC; **^#^** *P* < .05 versus WT‐TAC. Bars = 500 μm. **D‐H,** WGA‐stained section of left ventricles and quantification of myocyte cross‐sectional area (**D**), ANP mRNA expression (1.5‐fold at 4 weeks, 1.5‐fold at 8 weeks) (**E**), representative image of perivascular (**F**) and interestitital fibrosis (**G**) and quantification of fibrotic area, Col1A1mRNA expression (1.5‐fold at 4 weeks, 1.5‐fold at 8 weeks) (**H**) in WT and CKO mice before and after (4 and 8 weeks) TAC (n = 8 per group). *P < .05 versus mice before TAC; **^#^** *P* < .05 versus WT‐TAC. D, Bars = 50 μm; F and G, Bars = 100 μm. **I,** α‐SMA protein levels (1.3‐fold) in heart from WT and CKO mice before and post‐TAC 8 weeks (n = 4 per group). *P < .05 versus WT‐TAC. Statistical significance was determined by the two‐sided *t*‐test (B‐H) or Mann–Whitney *U* test (I)

To characterize the biological role of mir15a and mir16‐1 (mir15a/mir16‐1) in the adult heart, we developed a CM‐specific tamoxifen‐inducible mir15a/mir16‐1 knockout mouse (mir15a/mir16‐1 CKO) line by crossing mir15a/mir16‐1^flox/flox^ and Myh6‐mER‐Cre mice. mir15a/mir16‐1^fl/fl^ Myh6‐Cre^–^ mice served as control mice (WT). After tamoxifen administration, qRT‐PCR analysis confirmed an approximate 75% loss of mir15a/mir16‐1 expression in mir15a/mir16‐1 CKO mouse heart, but not in other organs (Figures S4A and S4B). Compared to WT mice, CKO mice exhibited no noticeable change in general morphology or baseline cardiac function before TAC. The mir15a/mir16‐1 CKO and WT mice were subjected to TAC operation after tamoxifen administration (Figure 2A). Serial echocardiography assessed left ventricular geometry and function in WT and CKO mice before and 2, 4, and 8 weeks post‐TAC. Parameters defining cardiac hypertrophy increased, and cardiac function (ejection fraction [EF]) decreased in CKO mice compared with WT mice after TAC (Figure 2B and Table S3). We also evaluated the echocardiography measurement in short‐axis and long‐axis in WT and CKO mice at 0 and 4 weeks of TAC. The echocardiographic parameters extracted from the short axis were similar to those from the long axis (Figure S5). CKO hearts harvested four and eight after TAC were significantly larger compared to control (Figure 2C). CKO mouse hearts exhibited increased heart weight to body weight and tibial length (HW/BW/TL) ratios (Figure 2C), increased expression of cardiac hypertrophy marker (atrial natriuretic peptide [ANP]), and increased CM size (Figures 2D and 2E). Myocardial fibrosis increased in CKO hearts compared to WT heart (evidenced by quantitative analysis of Masson's trichrome staining, Figures 2F and 2G). The mRNA levels of collagen I were increased in CKO mice at 4 and 8 weeks post‐TAC (Figure 2H). Finally, protein levels of α‐SMA (a marker of myofibroblasts) were increased in CKO mouse hearts compared to WT at 8 weeks post‐TAC (Figure 2I). To investigate if spontaneous hypertrophy occurred, we regularly monitored cardiac function in CKO mice and WT mice with aging. Importantly, CKO mice spontaneously developed concentric cardiac hypertrophy at 36 weeks of age with an increased left ventricular mass and IVS, and progressed to the heart failure at 42 weeks of age with a decreased EF (Figure S6 and Table S4). Thus, CM‐derived mir15a/mir16‐1 is an endogenous negative regulator of cardiac hypertrophy and heart failure induced by pressure overload, even aging.

### 3.3 Novel transcription factor C/EBPβ suppresses mir15a/mir16‐1 expression in the hypertrophic myocardium

**FIGURE 3:**
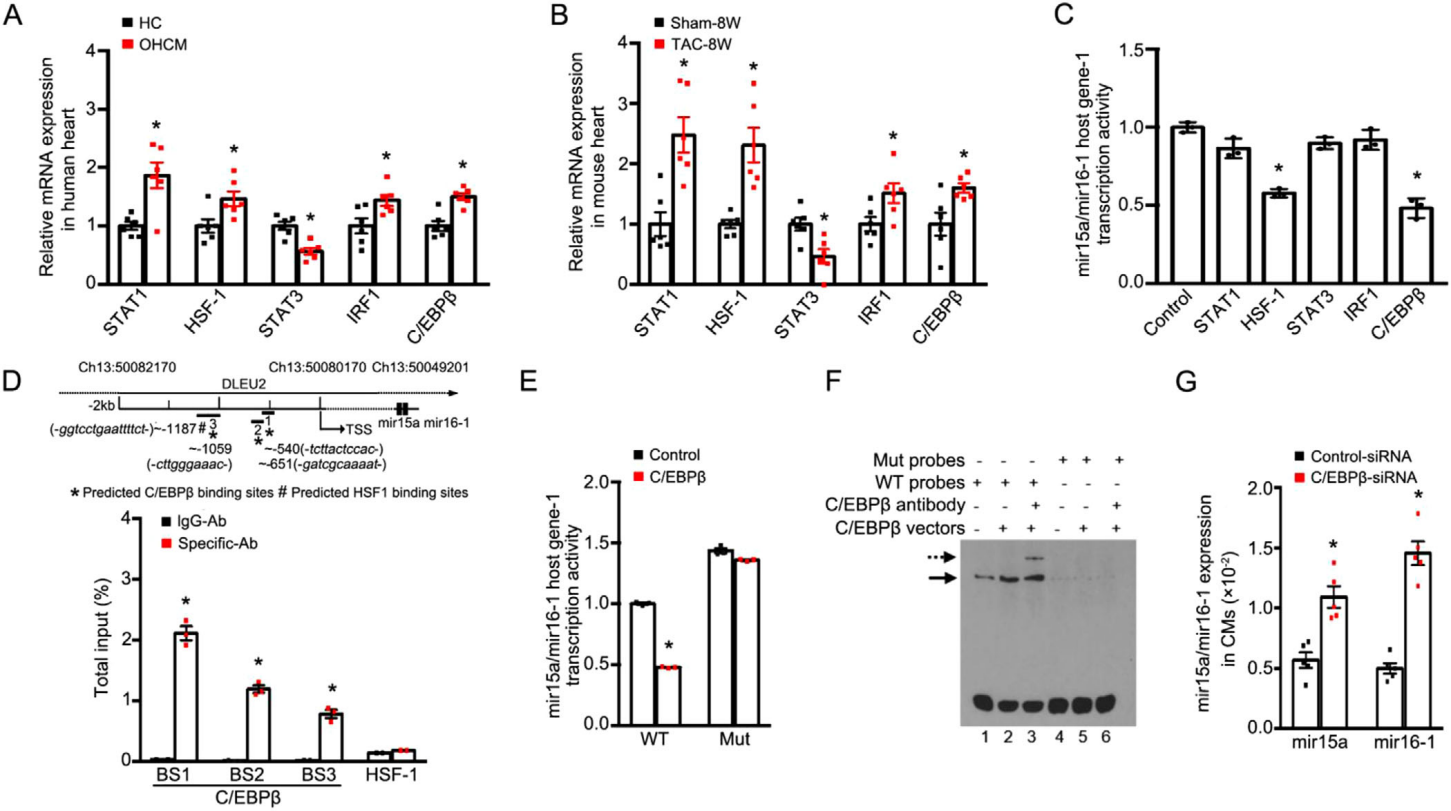
**Downregulation of mir15a/mir16‐1 cluster in cardiomyocytes by C/EBPβ. A,** qRT‐PCR indicated the expression of STAT1 (1.9‐fold), HSF1 (1.5‐fold), STAT3 (0.6‐fold), IRF1(1.4‐fold), and C/EBPβ (1.5‐fold) in human samples from representative normal control and hypertrophic hearts (n = 6 per group). *P < .05 versus HC. **B,** qRT‐PCR indicated the expression of STAT1 (2.5‐fold), HSF1 (2.3‐fold), STAT3 (0.5‐fold), IRF1(1.4‐fold), and C/EBPβ (1.6‐fold) in hearts from mice subjected to TAC (n = 6 per group). *P < .05 versus sham operation. **C,** Quantification of luciferase activity in cells with human pri‐mir15a/mir16‐1‐luciferase reporter constructs followed by TF overexpression as indicated (n = 3 experiments with 5 well replicates). *P < .05 versus control vector. **D,** Putative C/EBPβ or HSF1 binding sites are shown as asterisks or pound signs. Consensus sequences are italicized in parentheses. PCR amplicons 1, 2, and 3 are shown as numbered lines. Chromatin immunoprecipitation assays were performed using IgG, C/EBPβ, or HSF1 antibody (Ab) in cells stimulated with PE (n = 3 experiments). *P < .05 versus IgG Ab. **E,** Quantification of luciferase activity in cells transfected with wild‐type construct of pri‐mir15a/mir16‐1 or its mutant at the C/EBPβ‐binding site. Luciferase values normalized to the empty vector control (n = 3 experiments with 3 well replicates). *P < .05 versus control vector. **F,** EMSA assay showed specific binding complexes with 293T cells transfected with C/EBPβ expression vectors (lane 2). A weak signal was observed in non‐transfected 293T cells (lane1). Supershift experiments indicated C/EBPβ‐binding specificity (lane 3). Arrows indicate specific C/EBPβ‐DNA complexes (straight line) or antibody‐ C/EBPβ–DNA supershift complexes (dashed line). When the putative CEBP binding site was mutated, no DNA/protein complex was observed (lanes 4‐6). Bottom signals correspond to free probes. **G,** mir15a and mir16‐1 expression in CMs transfected with control‐siRNA or C/EBPβ‐siRNA subjected to PE (n = 5 per group). *P < .05 versus Control‐siRNA. Statistical significance was determined by the two‐sided *t*‐test (A, B, D, and G), by 1‐way ANOVA Tukey's post‐hoc test (C), by Mann‐Whitney test (E) Abbreviation: TSS, transcription start site.

Next, we investigated the regulation of mir15a/mir16‐1 expression. Primary mir15a/mir16‐1 is located in the intron of host genes (a long noncoding RNA named DLEU2, termed pri‐mir15a/mir16‐1).^13^, ^16^ Using bioinformatics analysis, we predicted 99 transcription factors (TFs) potentially regulating mir15a/mir16‐1 expression via binding of the pri‐mir15a/mir16‐1 promoter region. Utilizing previous established database (differently expressed TFs in the OHCM patient heart compared to normal control),^17^ 19 of 99 TFs were identified by Venn analysis. Employing qRT‐PCR analysis, we evaluated the expression of these 19 TFs in the normal and hypertrophic hearts from humans and the mouse TAC model. Among 19 TFs, four TFs (STAT1, HSF1, IRF1, and C/EBPβ) were consistently upregulated. One TF (signal transducer and activator of transcription 3 [STAT3]) was downregulated in both human and mouse hypertrophic hearts (Figures 3A and 3B, Figures S7A and S7B).

To determine whether five candidate TFs transcriptionally regulated mir15a/mir16‐1, we constructed luciferase reporter plasmids containing an upstream pri‐mir15a/mir16‐1 coding sequence. Luciferase reporter assays revealed that ectopic overexpression of C/EBPβ or HSF1 significantly decreased pri‐mir15a/mir16‐1 promoter activity (Figure 3C). We analyzed putative response elements of C/EBPβ or HSF1 in pri‐mir15a/mir16‐1, and performed a chromatin immunoprecipitation assay which demonstrated that C/EBPβ, but not HSF1, was enriched in the predicted binding sites of pri‐mir15a/mir16‐1 (Figure 3D). Minimal enrichment was detected in IgG isotype control experiments. We next generated a mutant construct containing the promoter region of pri‐mir15a/mir16‐1, with a mutated C/EBPβ‐binding site. C/EBPβ‐induced transcription inhibition was absent in cells transfected with mutant constructs (Figure 3E). Electrophoretic mobility shift assay experiment further confirmed that C/EBPβ bound the oligonucleotides with WT C/EBPβ‐binding sites from the pri‐mir15a/mir16‐1 promoter, whereas no binding was observed with mutant oligonucleotides. We observed a supershift with the C/EBPβ‐specific antibody with nuclear extracts containing C/EBPβ (Figure 3F). Finally, we evaluated whether C/EBPβ negatively regulated mir15a/mir16‐1 expression in CMs. Consistent with a previous report,^18^ phenylephrine (PE) treatment increased C/EBPβ expression in CMs (Figure S7C). Transfecting CMs with C/EBPβ‐specific siRNA restored the expression of mir15a/mir16‐1 when stimulated by PE (Figure 3G and Figure S7D). Collectively, these results provide strong evidence that C/EBPβ directly decreases mir15a/mir16‐1 at the transcript level in the hypertrophic myocardium.

### 3.4 Clarification of downstream signaling mediating the anti‐hypertrophy actions of mir15a/mir16‐1

**FIGURE 4:**
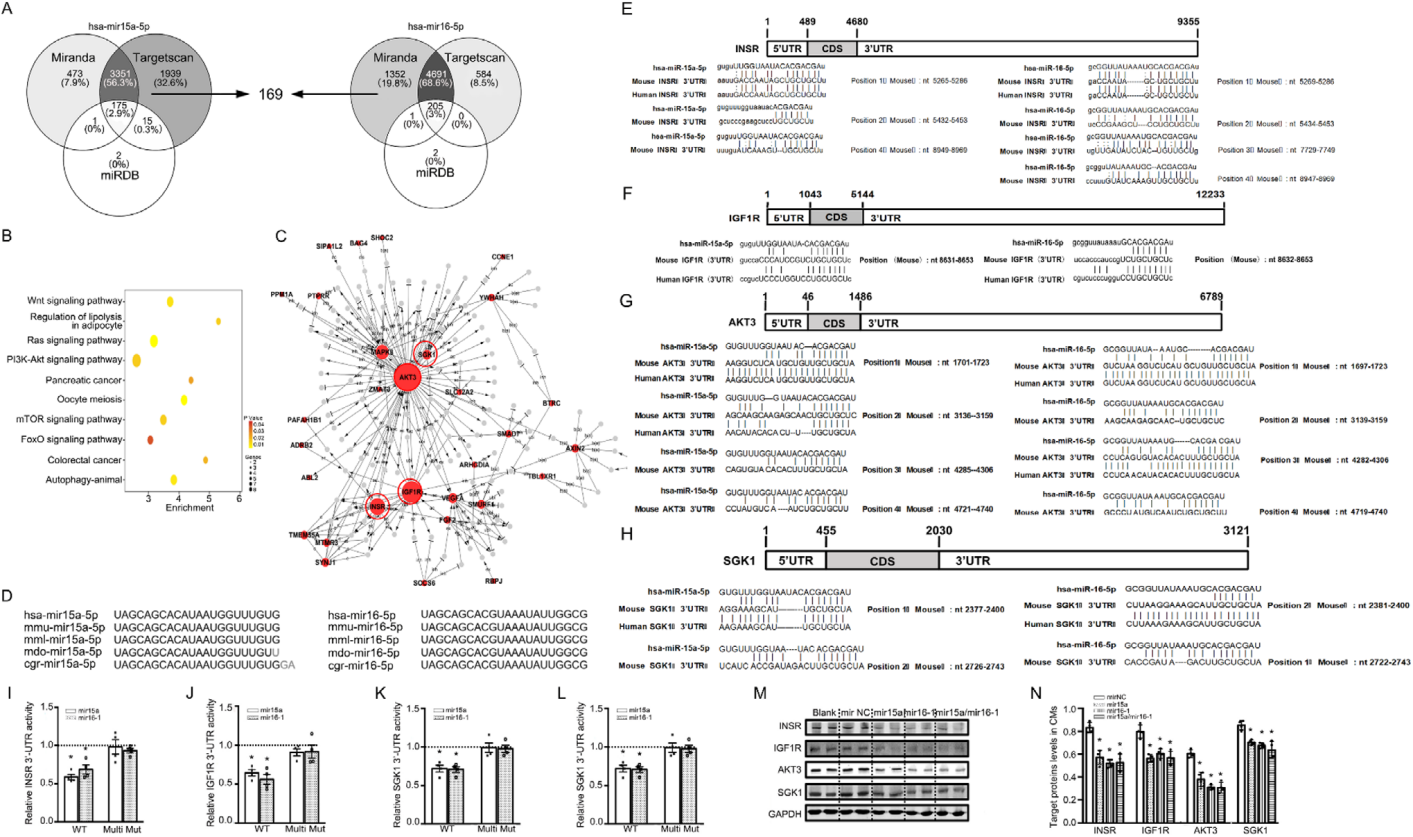
**mir15a/mir16‐1 targets multiple genes of insulin/IGF1 genes. A,** Venn intersection of putative target genes of hsa‐mir15a‐5p and has‐mir16‐5p, based upon three databases (Targetscan version 7.1, miRDB, and miRanda). **B,** Pathway analysis based on 169 putative targets genes. **C,** Putative target genes were connected in a network based on established protein‐protein interactions and signaling pathways. Nodes represent genes. Node area is the degree to which other genes interact with the given gene. **D,** Schematic representation of mir15a and mir16 sequence preservation in mammals. **E‐H,** Schematic diagram representing the potential binding sites for mir15a or mir16 in the 3′UTR of INSR, IGF1R, AKT3, and SGK1 in mice, and the corresponding binding sites in humans. **I‐L,** Luciferase reporters with wild‐type or mutant target gene‐UTR (INSR, IGF1R, AKT3, SGK1, respectively), were cotransfected with mimic‐mir15a, mimic‐mir16‐1, or mimic‐mir NC, respectively, then luciferase activity was determined. Values are presented as relative fold change of luciferase activity in mimic‐mir15a or mimic‐mir16‐1 transfection relative to luciferase activity in mimic‐mirNC transfection (n = 3 experiments with 3 replicates). *P < .05 versus mimic‐mirNC. **M and N,** Western blot expression analysis of INSR (0.7‐fold, 0.6‐fold, 0.6‐fold in mir15a, mir16‐1, mir15a/mir16‐1, respectively), IGF1R (0.7‐fold, 0.8‐fold, 0.7‐fold in mir15a, mir16‐1, mir15a/mir16‐1, respectively), AKT3 (0.6‐fold, 0.5‐fold, 0.5‐fold in mir15a, mir16‐1, mir15a/mir16‐1, respectively), SGK1 (0.8‐fold, 0.8‐fold, 0.8‐fold in mir15a, mir16‐1, mir15a/mir16‐1, respectively) expression in CMs untreated (blank) or treated with mimic‐mirNC, mimic‐mir15a, mimic‐mir16‐1, or mimic‐mir15a plus mimic‐mir16‐1 (n = 2 experiments with 2 well replicates). GAPDH levels served as loading control. *P < .05 versus mimic‐mirNC. Statistical significance was determined by the two‐sided *t*‐test (I‐L), by 1‐way ANOVA Tukey's post hoc test (N)

We next performed pathway analysis involving 169 overlapping putative target genes of mir15a and mir16‐1 in three databases (Targetscan version 7.1, miRDB, and miRanda, Figure 4A). Pathways involving putative mir15a/mir16‐1 targets included multiple pro‐hypertrophy pathways (PI3K‐serine/threonine kinase (AKT), mammalian target of rapamycin (mTOR), and FOXO, Figure 4B). To identify the key target genes, regulatory network maps were constructed from the 169 target genes. Notably, AKT3, insulin receptor (INSR), and insulin like growth factor 1 receptor (IGF1R) were the most significant genes per outdegree size, while serum/glucocorticoid regulated kinase 1 (SGK1) shared the greatest sequence homology with the AKT family^19^ (Figure 4C). mir15a and mir16‐1 sequences are highly conserved among mammals (Figure 4D). Analysis of the 3′‐untranslated region (UTR) of INSR, IGF1R, AKT3, and SGK1 revealed multiple potential binding sites for both mir15a and mir16‐1 (Figure 4E‐H). Mouse INSR, IGF1R, AKT3, and SGK1 as mir15a and mir16‐1 targets were validated by luciferase assays. Transfection of a plasmid containing the luciferase sequence of native INSR, IGF1R, AKT3, SGK1 3′‐UTR and a mir15a and mir16‐1 mimic significantly decreased normalized luciferase activity (Figure 4I‐L). Complete inhibition was observed when all binding positions were mutated (Figure 4I‐L). Western‐blot results supported the data obtained by reporter gene assays for INSR, IGF1R, AKT3, and SGK1 in CMs (Figures 4M and 4N). Similarly, the mir15a, mir16‐1, and the combination treatment also inhibited AKT3 protein levels in CM‐like AC16 cells (Figure S8).

**FIGURE 5:**
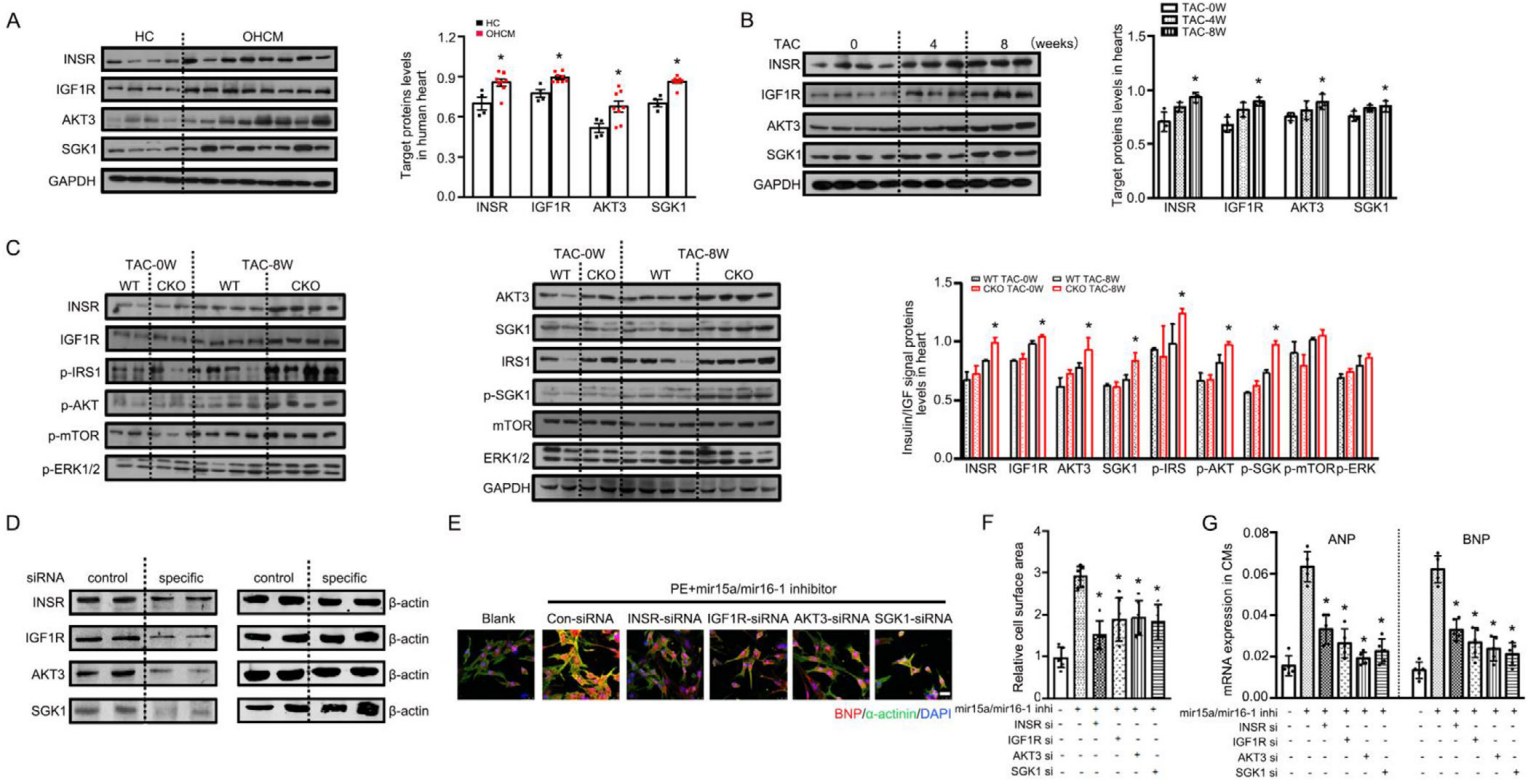
**Knockdown of mir15a/mir16‐1 hyperactivates insulin/IGF1 signaling. A,** Western blot analysis of target genes in human heart samples from representative healthy control (n = 4) and hypertrophic hearts (n = 8). Bar graphs indicate quantitative levels of INSR (1.2‐fold), IGF1R (1.2‐fold), AKT3 (1.3‐fold), SGK1 (1.2‐fold). *P < .05 versus HC. **B,** Western blot analysis of target genes in hearts before (0) (n = 4) and after 4 and 8 weeks TAC (n = 3). Bar graphs indicate quantitative levels of INSR (1.3‐fold at 8 weeks), IGF1R (1.3‐fold at 8 weeks), AKT3 (1.2‐fold at 8 weeks), SGK1 (1.2‐fold at 8 weeks). *P < .05 versus before TAC. **C,** Western blot analysis of insulin‐IGF1 signaling‐related proteins in hearts from WT and CKO mice before (n = 2 per group) and after 8 weeks of TAC (n = 4 per group). Bar graphs indicate quantitative levels of INSR (1.2‐fold), IGF1R (1.1‐fold), AKT3 (1.2‐fold), SGK1 (1.2‐fold), p‐IRS (1.3‐fold), p‐AKT (1.2‐fold at 8 weeks), p‐SGK (1.3‐fold at 8 weeks), p‐mTOR (1.1‐fold at 8 weeks), p‐ERK (1.1‐fold at 8 weeks). *P < .05 versus WT‐TAC 8W. **D,** Western blot analysis of proteins levels in CMs transfected with control‐siRNA or INSR, IGF1R, AKT3, SGK1‐siRNA subjected to mir15a/mir16‐1 inhibitor treatment (50 nM) (n = 2 experiments with 2 well replicates). **E‐G,** CMs were transfected with control, INSR, IGF1R, AKT3, or SGK1‐siRNA for 24 hours. CMs were then treated with mir15a/mir16‐1 inhibitor and PE for another 48 hours. Hypertrophy was assessed by morphological change (**E**, bar = 50 um), cell surface area measurement (**F**), and mRNA expression of ANP (0.5‐fold in INSR‐siRNA, 0.4‐fold in IGF1R‐siRNA, 0.3‐fold in AKT3‐siRNA, 0.4‐fold in SGK1‐siRNA) and BNP (0.5‐fold in INSR‐siRNA, 0.4‐fold in IGF1R‐siRNA, 0.4‐fold in AKT3‐siRNA, 0.3‐fold in SGK1‐siRNA) (**G**) (n = 5 experiments with 2 well replicates). *P < 0.05 versus control‐siRNA. Statistical significance was determined by the two‐sided *t*‐test (A and C) or by 1‐way ANOVA Tukey's post hoc test (B, F, and G)

We next determined the expression levels of four mir15a/mir16‐1 target genes in human and mouse hypertrophic hearts. Protein expression levels of INSR, IGF1R, AKT3, and SGK1 were significantly increased in hypertrophic hearts compared to control (Figures 5A and 5B). Activated INSR and IGF1R interact with insulin receptor substrate 1 (IRS1) to activate an intracellular signaling network, including AKT, mTOR, and mitogen‐activated protein kinase 1 (ERK1)/2.^20^, ^21^ We then analyzed insulin‐IGF1 signaling in the hearts of CKO and control mice post‐TAC. CKO hearts exhibited significantly increased expression of target protein, elevated IRS1/AKT/SGK phosphorylation, and slightly increased subsequent activation of ERK1/2 and mTOR (Figure 5C). These data confirmed INSR, IGF1R, AKT3, and SGK1 as novel targets of the mir15a/mir16‐1 cluster. The knockdown of mir15a/mir16‐1 increased the activation of insulin‐IGF1 signaling in the heart.

The results described above demonstrate that mir15a/mir16‐1 inhibition is sufficient to activate a hypertrophic signaling system. The mir15a/mir16‐1 inhibitor could promote CMs hypertrophy, indicated by an increase in CMs surface area and ANP/B‐type natriuretic peptide (BNP) expression (Figure S9A‐C). To further determine whether activation of this pathway is required for hypertrophic effect, we determined the effect of INSR, IGF1R, AKT3, or SGK1 knockdown (by transfection with their specific siRNA, Figure 5D) upon mir15a/mir16 inhibition‐induced hypertrophic response. The levels of four target genes were increased in CMs transfected with mir15a/mir16‐1 inhibitor compared to mir NC inhibitor (Figure S9D). CMs treated with mir15a/mir16‐1 inhibitor exhibited hypertrophic responses, as supported by morphological change (Figures 5E and 5F) and expression of hypertrophic genes (BNP and ANP) expression (Figure 5G). Importantly, the hypertrophic response was abolished by INSR, IGF1R, AKT3, or SGK1 knockdown (Figure 5E‐G). Similar to mRNA data, BNP protein levels were decreased in CMs when INSR/IGF1R/AKT3/SGK1 were blocked, respectively (Figure S9E). Taken together, these data suggest INSR, IGF1R, AKT3 and SGK1 are novel target genes contributing to mir15a/mir16‐1 inhibition‐mediated cardiac hypertrophy.

### 3.5 Evidence supporting mir15a/mir16‐1 as a novel therapeutic target against cardiac hypertrophy

**FIGURE 6:**
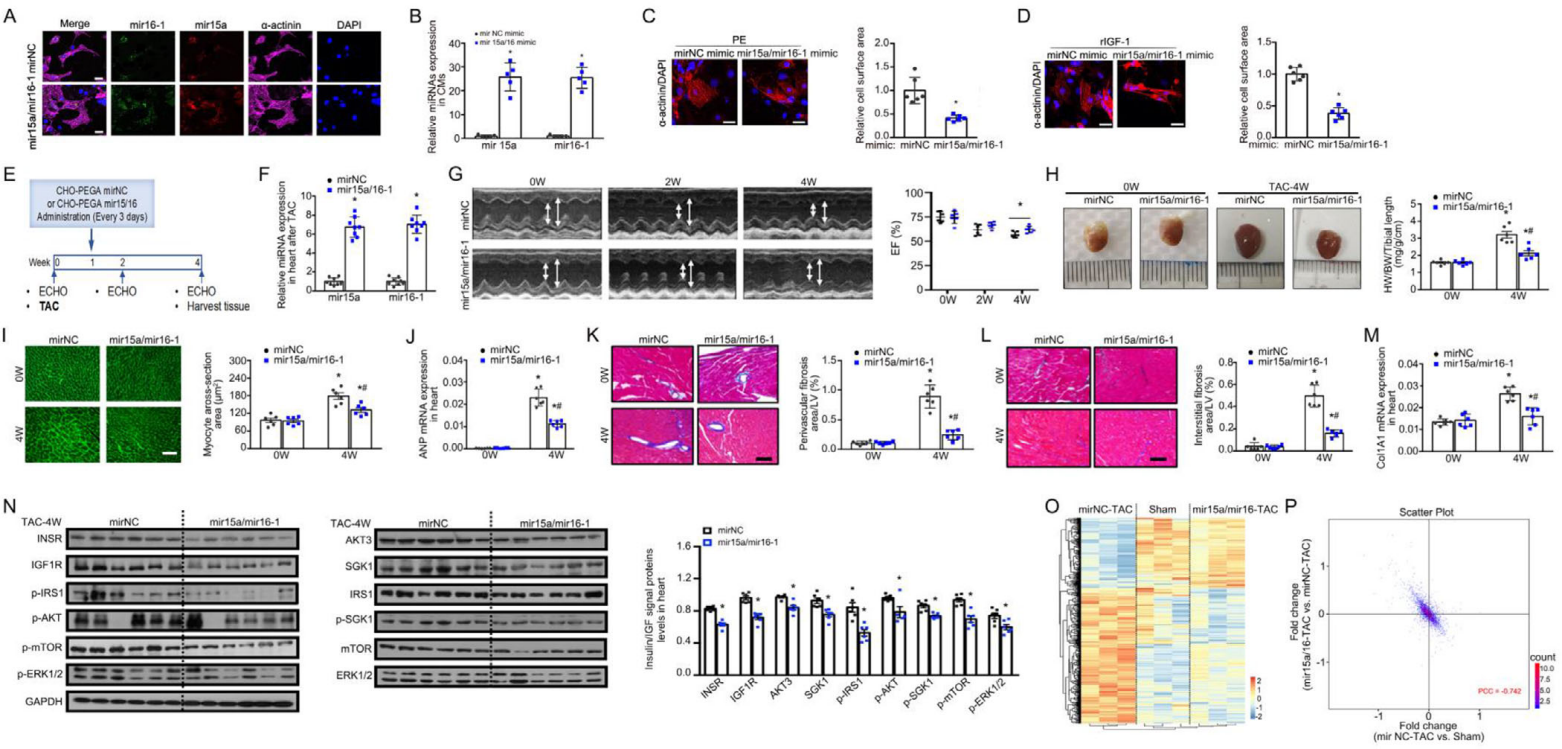
**Replenishment of mir15a/mir16‐1 protects against cardiac hypertrophy and failure. A,** In situ hybridization of mir15a and mir16‐1 in CMs transfected with CHO‐PGEA‐containing mirNC or mir15a/mir16‐1 for 24 hours. Scale bar = 25 μm. **B,** qRT‐PCR revealed mir15a and mir16‐1 expression in CMs transfected with CHO‐PGEA‐containing mirNC or mir15a/mir16‐1. *P < .05 versus CHO‐PGEA‐containing mirNC. **C and D,** CMs transfected with CHO‐PGEA‐containing mirNC or mir15a/mir16‐1 followed by PE at dosage of 100 nM (C) or recombinant murine IGF1(Peprotec, #250‐19) at dosage of 30 ng/mL (D) stimuli for 24 hours. Hypertrophy is assessed by sarcomere organization (bar = 25 um) and cell surface area measurement. *P < .05 versus CHO‐PGEA‐containing mirNC. **E,** Protocol for CHO‐PEGA‐containing mir15a/mir16‐1 therapy in the mouse TAC heart hypertrophy model. **F,** qRT‐PCR revealing cardiac mir15a and mir16‐1 expression in mice subjected to CHO‐PEGA‐mirNC or mir15a/mir16‐1 treatment for 4 weeks (n = 8 per group). *P < .05 versus CHO‐PEGA‐ mirNC. **G,** M‐mode echocardiographic imaging and EF analysis of mice treated with CHO‐PEGA‐mir NC or CHO‐PEGA‐mir 15a/mir16‐1 before and after 2 and 4 weeks TAC. ^#^ *P* < .05 versus CHO‐PEGA‐mir NC‐TAC. **H‐M,** Whole‐mount representation of heart and heart weight/body weight/tibia length ratios (**H**), WGA stained section of left ventricles and quantification of myocyte cross‐sectional area (**I**), ANP mRNA expression (0.5‐fold at 4 weeks) (**J**), representative image of perivascular (**K**) and interestitital fibrosis (**L**) and quantification of fibrotic area, Col1A1 mRNA expression (0.6‐fold at 4 weeks) (**M**) in mice treated with CHO‐PEGA‐mir NC or CHO‐PEGA‐mir15a/mir16‐1 before and after 4 weeks TAC (n = 6 per group). *P < .05 versus mice before TAC; **^#^** *P* < .05 versus CHO‐PEGA‐mir NC‐TAC. I, Bars = 50 μm; K and L, Bars = 100 μm. **N,** Western blot analysis of insulin‐IGF1 signaling‐related proteins in hearts from CHO‐PEGA‐mirNC or mir15a/mir16‐1 treated mice after 4 weeks of TAC. Bar graphs indicate quantitative levels of INSR (0.7‐fold), IGF1R (0.7‐fold), AKT3 (0.8‐fold), SGK1 (0.8‐fold), p‐IRS (0.2‐fold), p‐AKT (0.8‐fold), p‐SGK (0.9‐fold), p‐mTOR (0.7‐fold), p‐ERK (0.1‐fold) (n = 6 per group). *P < .05 versus CHO‐PEGA‐mir NC‐TAC. **O,** Heatmap demonstrating differentially expressed proteins among sham‐operation mice (Sham), TAC mice treated with either CHO‐PEGA‐mir NC (mir NC‐TAC), or mir15a/mir16‐1 (mir15a/mir16‐1‐TAC) (n = 3 per group). **P,** A representative scatter plot of protein expression fold‐change in mir15a/mir16‐TAC versus mir NC‐TAC (y‐axis) and mirNC‐TAC versus Sham (x‐axis). Each point represents a log2 (fold‐change) value for a protein. Statistical significance was determined by the two‐sided *t*‐test (B‐D and F‐N) Abbreviation: PCC, Pearson's correlation coefficient.

Previously, we showed that cholesterol‐terminated ethanolamine‐aminated poly glycidyl methacrylate (CHO‐PEGA) nanoparticles efficiently deliver miRNA into CMs and heart tissues.^22^ Employing the CHO‐PEGA carrier, we tested whether mir15a/mir16‐1 replenishment may rescue cardiac hypertrophy. CHO‐PEGA was utilized to deliver mir15a/mir16‐1 or negative control miRNA (mir NC) into cultured CMs after PE or recombinant IGF‐1 (rIGF‐1) treatment. Overexpression of mir15a/mir16‐1 in CMs transfected with the CHO‐PEGA‐mir15a/mir16‐1 complex was confirmed by both ISH and qRT‐PCR (Figures 6A and 6B). Furthermore, ectopic mir15a/mir16‐1 overexpression inhibited CM hypertrophy induced by PE or rIGF‐1 stimuli (Figures 6C and 6D).

WT mice were subjected to TAC, followed by intravenous injection of CHO‐PEGA‐mir15a/mir16‐1 or CHO‐PEGA‐mir NC (Figure 6E). Neither CHO‐PEGA‐mir15a/mir16‐1 nor CHO‐PEGA‐mir NC affected macroscopic and histological appearance of the sham‐operated mouse hearts. Robust induction of mir15a/mir16‐1 in mouse heart was verified by qRT‐PCR in animals treated with CHO‐PEGA containing‐mir15a/mir16‐1 4 weeks after TAC (Figure 6F). Echocardiography revealed that the CHO‐PEGA‐mir15/mir16 complex injection delayed the onset and progression of cardiac hypertrophy, as evidenced by decreased IVST. The CHO‐PEGA‐mir15/mir16 complex was able to attenuate LV dysfunction after TAC (Figure 6G and Table S5). After 4 weeks of TAC, the HW/BW/TL ratio significantly increased in mice injected with CHO‐PEGA‐mirNC, while CHO‐PEGA‐mir15/mir16‐1 treated animals manifested a blunted hypertrophic response (Figure 6H). Increased CM cell surface area, fibrosis, and the expression of hypertrophic /fibrotic genes (ANP/Col1A1) were significantly attenuated in the CHO‐PEGA‐mir15/mir16‐1 treated animals compared to CHO‐PEGA‐mir NC (Figure 6I‐M). CHO‐PEGA‐mir15a/mir16‐1 treatment significantly inhibited both the expression of mir15a/mir16‐1 target protein and insulin‐IGF1 signaling (Figure 6N).

To characterize the global impact of mir15a/mir16‐1 treatment on cardiac hypertrophy, we compared the protein profiles of sham‐operated mice (Sham), and TAC mice treated with CHO‐PEGA‐mir NC (TAC‐mir NC) or CHO‐PEGA‐mir 15a/mir16‐1 (TAC‐mir15a/mir16‐1). In total, 455 and 381 differentially expressed proteins (DEPs) were identified in TAC‐mir NC versus Sham or TAC‐mir15a/mir16‐1 versus TAC‐mir NC (filtering criteria: *P* < .05; fold change ≥1.2) by proteomic analysis, respectively (Figure 6O). The fold change of DEPs in TAC‐mir NC versus Sham and TAC‐mir15a/mir16‐1 versus TAC‐mir NC was highly negative correlated (Pearson's correlation coefficient = −0.742), indicating that replenishment of mir15a/mir16‐1 reversed the protein profile induced by pressure overload (Figure 6P). Taken together, these results provide proof‐of‐principle evidence that mir15a/mir16‐1 may serve as a novel therapeutic treatment for cardiac hypertrophy and dysfunction.

### 3.6 Circulating mir15a/mir16‐1 levels may predict cardiac hypertrophy in hypertensive patients

**FIGURE 7:**
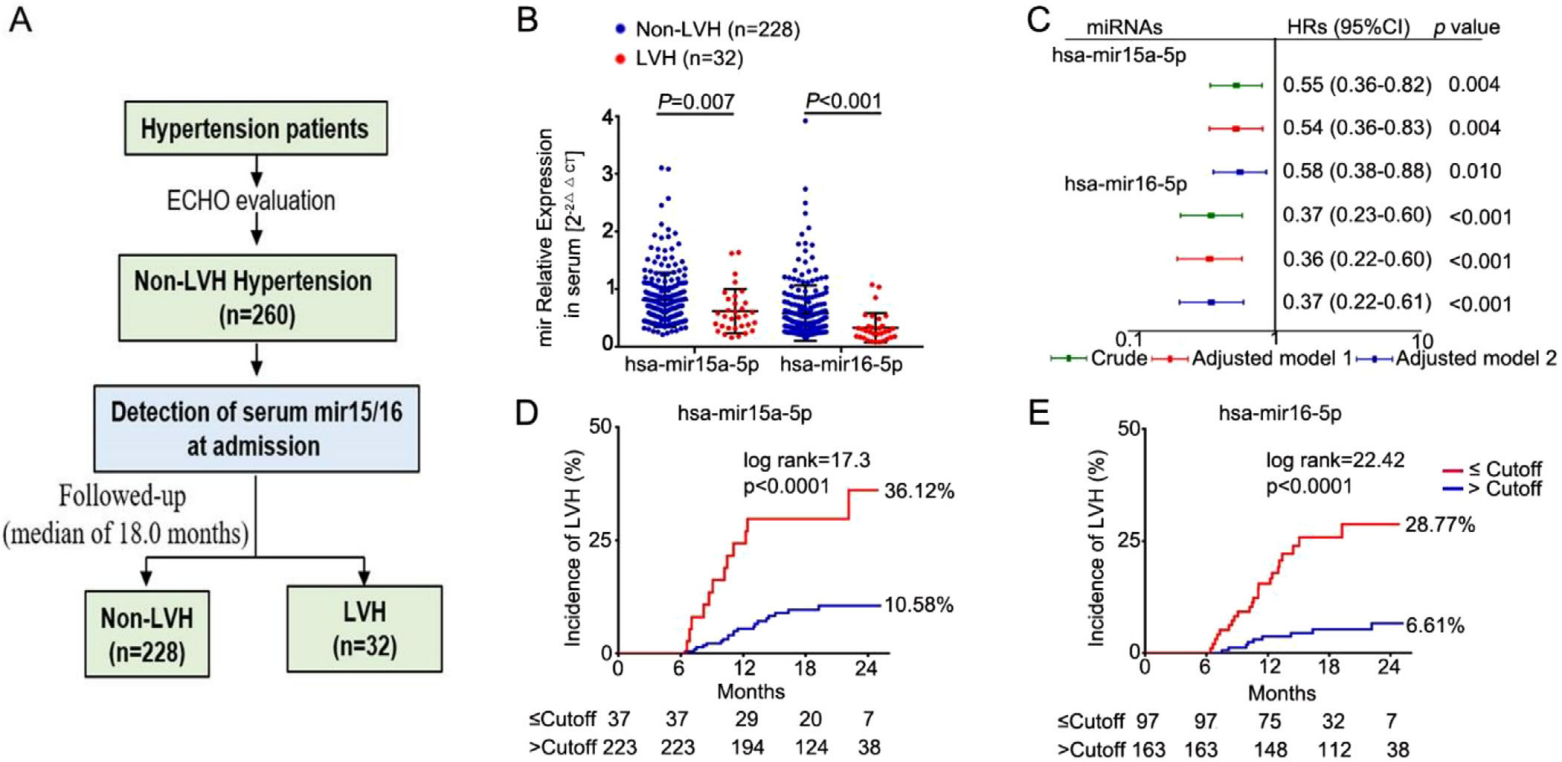
**Decreased mir15a/mir16‐1 levels associated with LVH in hypertensive patients. A,** Flow diagram for assessing the predictive value of serum mir15a‐5p and mir16‐5p for LVH incidence in hypertensive patients. **B,** qRT‐PCR analysis of mir15a‐5p and mir16‐5p expression at admission in hypertensive patients with (n = 32) or without (n = 228) incidental LVH during follow‐up. **C,** Univariate and multivariate Cox regression analyses of mir15a‐5p and mir16‐5p levels for incident LVH. Model 1 was adjusted for diabetes mellitus, coronary heart disease, body mass index, and estimated glomerular filtration rate, and LV mass index at admission; model 2 was adjusted for medication treatment of β‐blockers, angiotensin‐converting enzyme inhibitor/angiotensin receptor blocker, calcium antagonists, and diuretics. **D and E,** The prognostic values of mir15a‐5p or mir16‐5p levels for LVH were determined by Kaplan‐Meier and log‐rank test. Statistical significance was determined by the two‐sided *t*‐test **(B)**, by Cox regression analysis **(C)**, by log‐rank test **(D and E)**

Having demonstrated that mir15a/mir16‐1 downregulation aggravated cardiac hypertrophy in the pressure overload mouse model, we next determined the potential clinical application of our experimental findings. We studied the association of circulating mir15a‐5p and mir16‐5p levels with the progression of LVH in a small cohort of hypertensive patients without LVH at baseline (Figure 7A). Patient characteristics are depicted in Table S6. During a median of 18‐month follow‐up, 32 patients (12.3%) developed LVH. Patients with LVH exhibited significantly lower levels of mir15a‐5p (*P* = .007) and mir16‐5p (*P* < .001) than patients without LVH (Figure 7B). This decreased expression was unrelated to the gender, diabetes, coronary heart disease, or obesity (Figure S10A‐D). COX regression analysis was employed to estimate the likelihood of LVH with mir15a‐5p/mir16‐5p. Decreased mir15a‐5p (hazard ratio [HR] 0.55, 95% CI [0.36‐0.82], *P* = 0.004) and mir16‐5p ([HR] 0.37, 95% CI [0.23‐0.60], *P* < .001) were significantly associated with LVH risk, even after multivariable adjustment for important clinical characteristics (Figure 7C). Figures 7D and 7E illustrate LVH occurrence according to mir15a‐5p or mir16‐5p cutoff values. Patients with decreased mir15a‐5p or mir16‐5p levels were more likely to exhibit LVH during follow‐up. Serum mir15a‐5p and mir16‐5p levels may therefore serve as valuable biomarkers for LVH prediction.

## 4 DISCUSSION

In the present translational study, we make an observation in a human disease population with HCM, and confirm the causal relationship in a mouse model with pressure overload and in vitro experiments, and corroborate the findings in human patients. We identify that mir15a/mir16‐1 levels are negatively correlated with hypertrophic severity in HCM patients. Hypertrophic stress downregulates mir15a/mir16‐1 transcription via a C/EBPβ‐dependent mechanism. We combine in vivo and in vitro studies to demonstrate that miR15a/mir16‐1 represses insulin/IGF1 signaling, preventing cardiac hypertrophy and dysfunction development. Decreased mir15a/mir16‐1 levels are independently associated with LVH occurrence in patients. Our results suggest that mir15a/mir16‐1 cluster is a novel biomarker and anti‐hypertrophic regulator during hypertrophic heart failure, with promise as a therapeutic target.

We identify that mir15a/mir16‐1 is produced primarily by CMs. Its expression is significantly decreased in both human and mouse hypertrophic hearts. Decreased mir15a/mir16‐1 production during hypertrophic stress decreases cardiac mir15a/mir16‐1 release into circulation. Circulating mir15a/mir16‐1 levels are significantly decreased in HCM patients compared to HC, and are negatively correlated with hypertrophic severity. Importantly, patients with low mir15a/mir16‐1 levels were at high risk for the occurrence of LVH. LVH is an important marker of target organ damage in hypertension, and precedes the incidence of adverse cardiac events.^23^, ^24^ Identification of a biomarker with a predictive value for LVH in hypertensive patients is important, because it is not regularly identified whether LVH develops during any given antihypertensive regimen. These clinical results provide strong evidence underlining the association of decreased mir15a/mir16‐1 with pathological cardiac hypertrophy.

The regulatory mechanism of mir15a/mir16‐1 expression is unknown. We have provided consistent evidence identifying C/EBPβ as an inhibitory TF regulating mir15a/mir16‐1 in CMs. Surprisingly, STAT3, reportedly upstream of mir16,^25^ did not affect the promoter activity of mir15a/mir16‐1. Previous studies only reported increased mir16 expression during STAT3 knockdown, not providing evidence that STAT3 directly regulates mir15a/mir16‐1 at the transcript level.^25^, ^26^ C/EBPβ, a leucine zipper transcription factor, is pivotal in the regulation of heart development^27^ and physiological^28^ and pathological cardiac hypertrophy.^18^ Of interest, a constitutive upregulation of C/EBPβ has been described in PE‐treated CMs, as well as in hearts subjected to pressure overload.^18^ We observe increased C/EBPβ in hypertrophic hearts of HCM patients. C/EBPβ knockout in CMs produced a phenotype similar to that of anti‐hypertrophy,^18^ as observed in mir15a/mir16‐1 overexpressed CMs. Mice heterozygous for C/EBPβ (C/EBPβ^+/–^) developed cardiac hypertrophy to lesser extent, and are resistant to cardiac dysfunction in response to pressure overload or pregnancy stress compared to WT.^18^, ^28^ Some cardiac phenotypes seen in C/EBPβ^+/–^ mice may derive from upregulated mir15a/mir16‐1 expression. Our results implicate involvement of the C/EBPβ‐mir15a/mir16‐1 axis in pathologic hypertrophy development.

We provide a concrete causative link between downregulated mir15a/mir16‐1 and cardiac hypertrophy development. Pressure overload‐induced cardiac hypertrophy was exacerbated by CM‐specific mir15a/mir16‐1 knockdown, strongly suggesting that mir15a/mir16‐1 is an essential protector of cardiac hypertrophy. Consistent with this, replenishment of mir15a/mir16‐1, using nanoparticle‐carried miRNAs in mice subjected to TAC, shows the decreased cardiac hypertrophy and cardiac function improvement. We provide the compelling evidence that multiple key components of insulin‐IGF1 signaling (including INSR, IGF1R, AKT3, and SGK1) are targeted by mir15a/mir16‐1. It is interesting that the identified multiple targets of mir15a/mir16‐1 form a cooperative network regulating hypertrophy. Upon binding to their ligands, insulin and IGF1 receptors undergo auto‐phosphorylation, which in turn interact with IRS1/2 proteins and other binding partners to activate the PI3K‐AKT signaling pathway. SGK is highly homologous to AKT, sharing similar upstream activators and downstream targets. Both AKT and SGK can regulate the pro‐growth mTOR pathway. In turn, mTORC2 phosphorylates AKT at hydrophobic motif S473, and mammalian target of rapamycin complex 1 (mTORC1) phosphorylates SGK at the hydrophobic motif S422, leading to their full activation.^29^ Studies using pressure overload or spontaneously hypertension model, reported that the activation of mTOR signaling is essential for cardiac hypertrophy.^30^, ^31^ Overexpression of IGF1R,^32^ AKT3,^33^ and SGK1^34^ was also associated with marked cardiac hypertrophy and dysfunction.

Moreover, in response to mechanical overload, pathological processes, including CMs loss and, impaired fatty acid utilization, promoted the transition from compensated hypertrophy to contractile dysfunction and heart failure.^35^, ^36^ In this study, proteomics revealed that multiple myocardial fatty acid oxidation (MFAO)‐related pathways (i.e. fatty acid degradation/metabolism/elongation) were significantly downregulated in TAC‐induced hearts, a pattern similar to previous reports.^37^ Importantly, decreased MFAO‐related pathways were restored after replenishment with mir15a/mir16‐1 (Figure S11). Thus, mir15a/mir16‐1 CKO mice exhibited increased compensated hypertrophy at 4 weeks and decreased LV systolic function at 8 weeks after TAC, which might indicate that mir15a/mir16‐1 knockdown not only aggregated cardiac hypertrophy but also accelerated the progression from compensated hypertrophy into HF. In addition, we and others demonstrated that downregulated mir15a/mir16‐1 promoted cardiac fibrosis in the pathological condition.^38^ Although current identified target genes did not directly regulate fibrosis, previous study reported that mir15 family inhibited the pro‐fibrotic pathway by targeting TGFBR1 and several other genes within transforming growth factor (TGF)β pathway (including SMAD Family Member 3 [SMAD3]/7/8).^39^ Thus, the multiple targets of mir15a/mir16‐1 form a cooperative network for regulating hypertrophy, decompensated transition of hypertrophy, and cardiac fibrosis.

Despite the exciting current findings that the mir15a/mir16‐1 cluster serves to negatively regulate pathological cardiac hypertrophy, there are several limitations in our study that warrant further discussion and which will require future follow‐up studies. First, we found the association between mir15a/mir16‐1 cluster and cardiac hypertrophy in HCM patients, but tested its pathological role in the animal model of pressure overload‐induced cardiac hypertrophy. However, it is worth noting that the intermediary molecular events involved in HCM, including calcineurin, MAPK and TGFβ pathways are also activated in pressure overload‐induced cardiac hypertrophy.^40^ Second, it should be noted that mir15a/mir16‐1 modulate angiogenesis. Previous studies from the Emanueli C group revealed that mir15a/mir16‐1 inhibited neovascularization in limb ischemia by impairing the survival/migration of circulating proangiogenic cells and the expression of angiopoietins.^41^, ^42^ The miRNAs are known to work in a context‐dependent manner and in different scenarios. The mir15a/mir16‐1 expression appeared altered in different heart disease setting, upregulation in ischemic myocardium and, downregulation in hypertrophic myocardium. The potential suppressed angiogenetic response due to the excessive overexpression of mir15a/mir16‐1 may need to be avoided. Finally, the mir15a/mir16‐1 bio‐distribution in the internal organs showed the high accumulations in the heart, liver, and kidney after CHO‐PEGA‐mir15a/mir16‐1 treatment (Figure S12), suggesting that system delivery of CHO‐PEGA‐ mir15a/mir16‐1 could achieve cardiac enrichment but lacked cardiac‐specific ability. Thus, cardiac‐targeting delivery requires further study.

In conclusion, we provide the first evidence that circulating mir15a/mir16‐1 levels negatively correlate with hypertrophic severity and predict the occurrence of LVH. mir15a/mir16‐1 protects against pathological cardiac hypertrophy and sequelae (e.g. heart failure) by repressing insulin/IGF1 signaling. Nanoparticle‐carrying mir15a/mir16‐1 treatment decreases cardiac hypertrophy and improves cardiac function in an experimental animal model of pressure overload. Our study exhibits important clinical implications for both the treatment and prediction of cardiac hypertrophy.

## CONFLICT OF INTEREST

The authors declare that there is no conflict of interest that could be perceived as prejudicing the impartiality of the research reported

## AUTHOR CONTRIBUTIONS

Yulin Li, Jie Du, and Xin‐liang Ma contributed to the study design and data interpretation and wrote the manuscript. Hongchang Guo, Ke Ma, and Ping Li were responsible for the study of patients. Yulin Li, Wenjing Hao, Yao Jiao, and Jing Chen performed the experiments in animals and cells and contributed to data analysis and interpretation. Chen Xu and Fu‐jian Xu developed the nanoparticle‐carrying miRNAsystem. Wayne Bond Lau helped in writing the manuscript.

## DATA AND MATERIALS AVAILABILITY

All data are included in the manuscript or in the supporting materials.

## Supporting information

Supporting Information

## Data Availability

The data for the current analysis are available upon reasonable request to the corresponding author.
