# CORRIGENDUM

**DOI:** 10.1002/ctm2.302

**Published:** 2021-01-24

In Guo et al.^1^ the following errors were published in Figures 2, 4, and 6.

The ECHO image of WT, 4W in Figure 2B was a duplicate of CKO, 2W. The corrected Figure 2 is below:


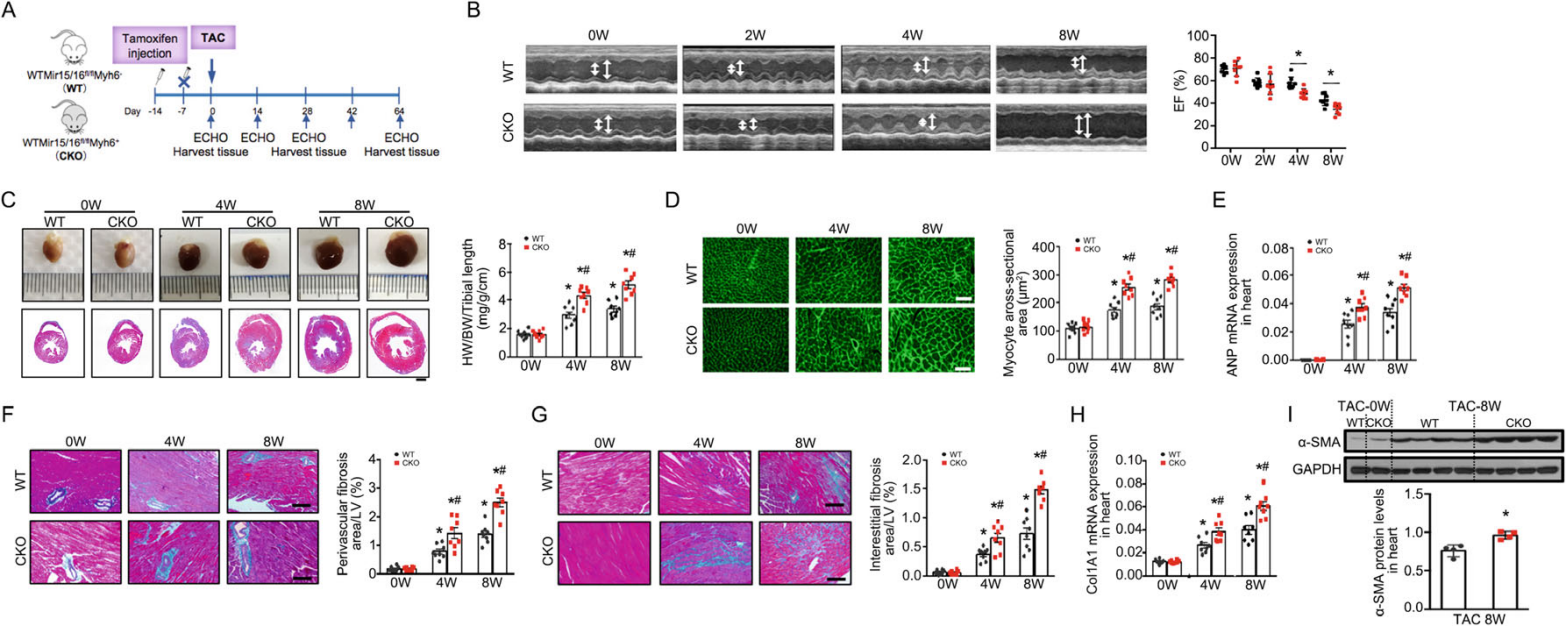


The bar graph in Figure 4K is a duplicate of Figure 4L. The corrected Figure 4 is below:


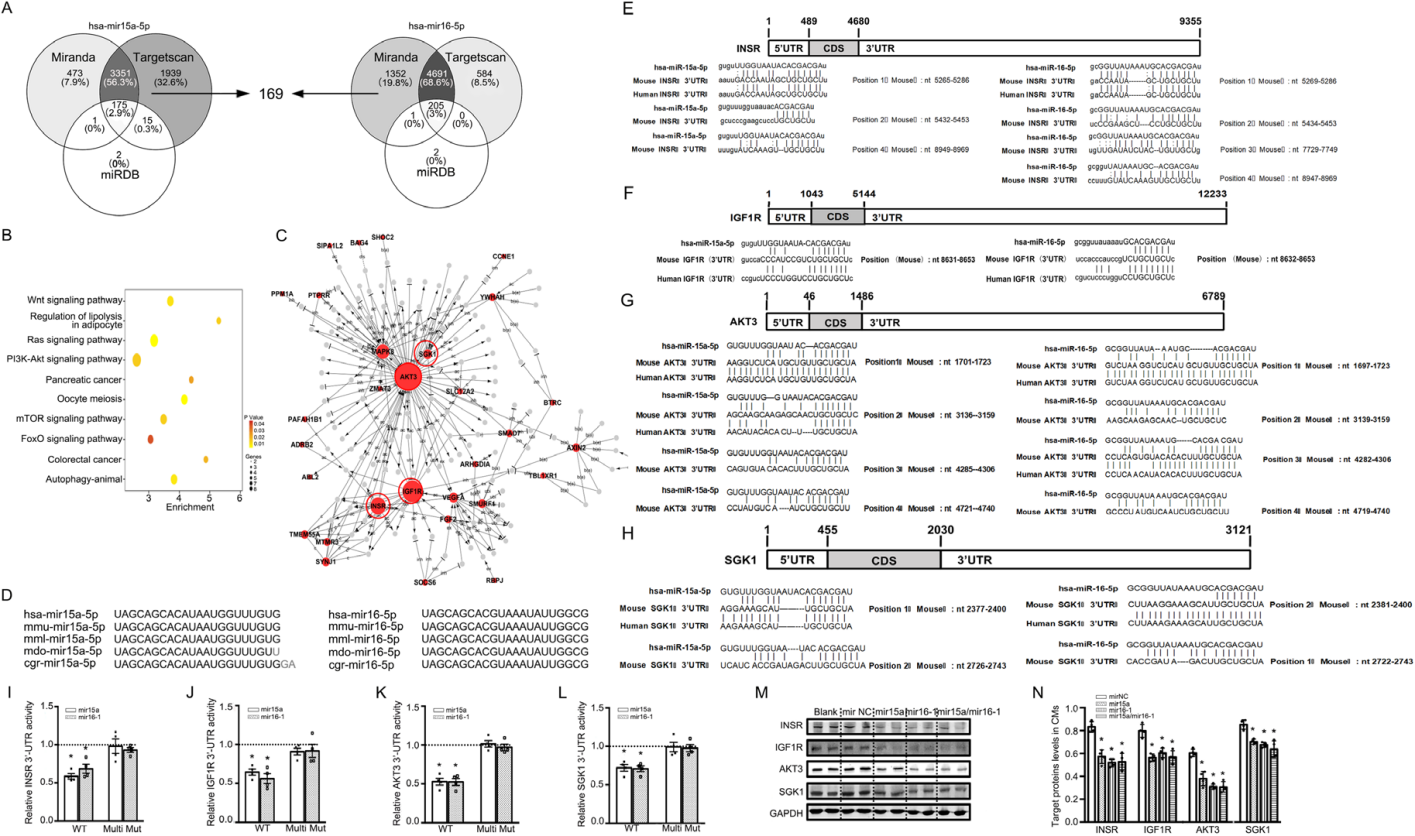


The ECHO image of mirNC, 2W in Figure 6G was a duplicate of mirNC, 4W. The corrected Figure 6 is below:


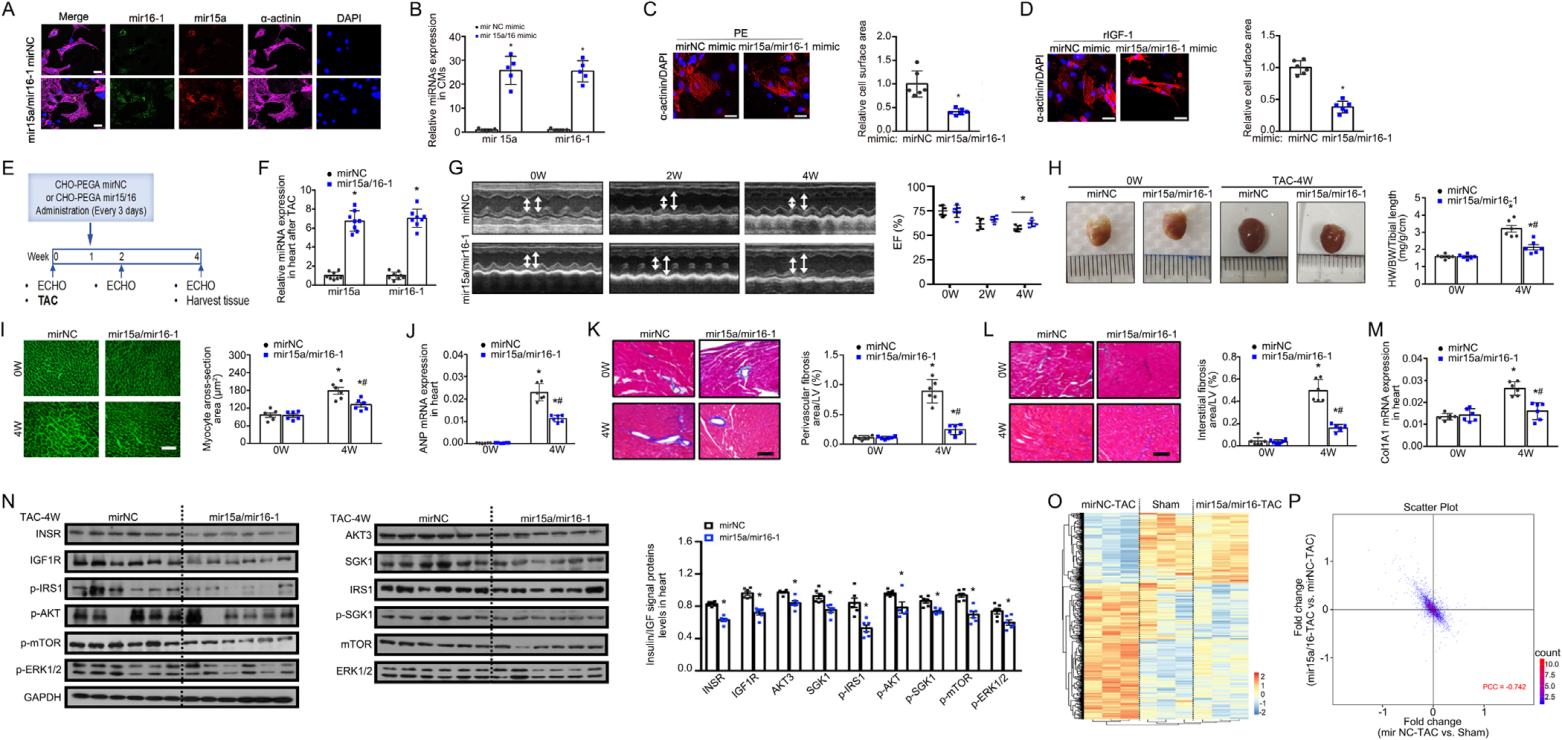


The online version of this article has been corrected.

The authors regret these errors.
