# Supplementary material for: mir15a/mir16‐1 cluster and its novel targeting molecules negatively regulate cardiac hypertrophy
Source: Clin Transl Med. 2020 Dec 15;10(8):e242. doi: 10.1002/ctm2.242 (PMC7737755; doi:10.1002/ctm2.242)
Supplement: Supplementary file 1 — Supporting Information [file CTM2-10-e242-s001.docx]

**Table of contents**

- **Supplementary Methods**
- **Supplementary figure 1**
- **Supplementary figure 2**
- **Supplementary figure 3**
- **Supplementary figure 4**
- **Supplementary figure 5**
- **Supplementary figure 6**
- **Supplementary figure 7**
- **Supplementary figure 8**
- **Supplementary figure 9**
- **Supplementary figure 10**
- **Supplementary figure 11**
- **Supplementary figure 12**
- **Supplementary table 1**
- **Supplementary table 2**
- **Supplementary table 3**
- **Supplementary table 4**
- **Supplementary table 5**
- **Supplementary table 6**

**Supplementary Methods**

**Study population**

Hypertrophic cardiomyopathy (HCM) population

The 93 patients with HCM were enrolled at Anzhen Hospital of Capital Medical University (Beijing, China). The diagnostic criteria of HCM were according to the recent European guidelines for the diagnosis and management of hypertrophic cardiomyopathies^1^ as follows: presence of a hypertrophic cardiac septum (≥15 mm) or combined presence of a hypertrophic cardiac septum (≥13 mm) and a positive family history. Obstructive HCM (OHCM) was defined as a peak Doppler left ventricular outflow tract pressure gradient (LVOT PG)>30 mm Hg. Healthy controls (HC) were also enrolled at Anzhen Hospital and matched for age and sex. All ultrasound examinations were performed using a commercially available echocardiographic machine with an S3 transducer (Vivid Seven System; GE Healthcare, Horten, Norway). The maximal wall thickness of the interventricular septum (IVS), left ventricular diameter, left ventricular end-diastolic diameter, end-systolic diameter was measured in M-mode images according to American Society of Echocardiography guidelines. The LVOT PG was calculated at rest with the simplified Bernoulli’s equation. Information on demographic characteristics, history, clinical presentation, physical examination, imaging information, and management was obtained from the medical records. CLINICAL TRIAL REGISTRATION: URL: <http://clinicaltrials.gov>. “An Integrative-"Omics" Study of Cardiomyopathy Patients for Diagnosis and Prognosis in China (AOCC)”, Unique identifier: NCT03076580.

Hypertension population

Hypertension patients were admitted to Anzhen Hospital. Inclusion criteria for the present study were ultrasound evidence of normal left ventricular (LV) mass index (LVMi) at the time of first clinical examination, no secondary forms of hypertension, age≥18 years. The study population consisted of 260 treated hypertensive patients. Exclusion criteria included significant valvular heart diseases, active myocarditis, recent acute coronary syndrome, chronic heart failure (defined as LV ejection fraction (EF)<50%), chronic kidney disease $\leq stage 3$(defined as glomerular filtration rate (GFR)>30 ml/min per 1.73 m^2^). Follow-up period was considered from the initial visit to the last available clinical assessment or the time of first evidence of LV hypertrophy (LVH). LVMi was calculated from a necropsy-validate formula and normalized for height in meters to the power of 2.7 (g/m^2.7^)^2^. LVH was defined as LVMi ≥50 g/m^2.7^ in men or ≥47 g/m^2.7^ in women^3^. Information on demographic characteristics, history, clinical presentation, physical examination, imaging information, and management was obtained from the medical records. After the initial hospitalization, patients were followed up for a median of 18 months. The outcomes were obtained by reviewing the medical records and by contacting each patient or their relatives individually. CLINICAL TRIAL REGISTRATION: URL: <http://clinicaltrials.gov>. “Prognostic Value of Plasma Biomarkers Among Patients With Hypertension (BIOMS-HTN)”, Unique identifier: NCT04107584.

**Human sample collection**

Human hypertrophic heart tissue samples were obtained from patients diagnosed with OHCM, undergoing septal myotomy surgery. Control samples were obtained from normal heart donor left ventricles. All applicable protocols involving the collection or use of patient materials respected the Declaration of Helsinki. All heart specimens were processed immediately after surgery and stored at less than -80°C, until total RNA and protein were extracted. Blood samples were collected from participants and drawn into sterile polyolefin resin tubes with coagulant. The samples were centrifuged at 2000$\times$g for 10 min at room temperature. The supernatant serum was quickly removed, aliquoted in a RNase-free microfuge tubes, and stored at −80°C.

**Serum RNA isolation and miRNA-sequencing**

Total serum RNA was extracted using the miRVana miRNA isolation kit by following the manufacturer’s protocol (Ambion, Thermo Scientific, Wilmington, DE, USA). Each RNA sample was quantified with a spectrophotometer (NanoDrop 1000, Thermo Scientific, Wilmington, DE, USA). Total RNA of each sample was used to prepare the miRNA sequencing, which included the following steps: 1) 3'-adaptor ligation; 2) 5'-adaptor ligation; 3) cDNA synthesis; 4) PCR amplification; and 5) size selection of approximately 135–155 bp of PCR-amplified fragments (corresponding to 15–35 nt of small RNAs). The libraries were denatured as single-stranded DNA molecules, captured on Illumina flow cells, amplified in situ as clusters, and finally sequenced for 36 cycles on the NextSeq 500 (Illumina, San Diego, CA, USA) per the manufacturer’s instructions.

**Exosomes isolation**

The plasma from patients with HCM and health control were diluted with an equal volume of PBS, centrifuged at 2,000 g for 30 min at 4℃ and then the supernatant was collected and centrifuged at 12,000 g for 45 min at 4℃ to eliminate cell and cell debris contamination. The supernatant was further centrifuged at 110,000g (Beckman, OptimaTM MAX-XP Ultracentrifuge, USA) for 120 min at 4 °C. The pellets were resuspended with PBS, filtered with 0.2 μm filter and the filtered solution was centrifuged at 110,000g for 70 min at 4 °C again. The exosome pellet was ready for qRT-PCR analysis.

**qRT-PCR analysis of serum miRNAs**

miRNA expression was assessed by qRT-PCR. We spiked 100 pM of synthetic cel-miR-40-3p into serum RNA. Isolated RNA was converted to complementary DNA (cDNA) with the TaqMan miRNA Reverse Transcription Kit (Life Technologies, Eugene, OR, USA). TaqMan (miR-16-5p, miR-15a-5p, miR-192-5p, miR-342-3p, miR-151a-3p, miR-98-5p, miR-340-5p, miR-99b-5p, and let-7c-5p), RT primers, and 2 ng of total RNA from each sample was reverse-transcribed by following the manufacturer’s protocol (Applied Biosystems, Foster City, CA, USA). The quantitative real-time polymerase chain reaction (qRT-PCR) was conducted in 96-well plates with RT product and TaqMan PCR master mix and TaqMan probes for each miRNA by the ABI Prism Model 7900 HT instrument (Applied Biosystems, Foster City, CA, USA). The qRT-PCR was performed in duplicate for all samples. The 2 –ΔΔCt method was used to analyze the relative changes in miRNA expression^4^. Expression levels of miRNAs were normalized to a nonendogenous synthetic miRNA. The PCR primers for miRNA are shown in the table below:

hsa-miR-99b-5p: 5’GGCACCCGTAGAACCGA3’, 5'CAGTGCGTGTCGTGGAGT3';

hsa-miR-342-3p: 5’GGGTCTCACACAGAAATCG3’, 5'CAGTGCGTGTCGTGGAGT3';

hsa-miR-15a-5p: 5’GGGTAGCAGCACATAATGG3’, 5'CAGTGCGTGTCGTGGAGT3';

hsa-miR-16-5p: 5’GGGTAGCAGCACGTAAATA3’, 5'CAGTGCGTGTCGTGGAGT3’;

hsa-miR-340-5p: 5'GCGGTTATAAAGCAATGAGA3’, 5’GTGCGTGTCGTGGAGTCG3’;

hsa-miR-192-5p: 5’GGGGCTGACCTATGAATTG3', 5'CAGTGCGTGTCGTGGAGT3';

hsa-let-7c-5p: 5'TGCGTGTCGTGGAGTC3', 5'TGCGTGTCGTGGAGTC3';

hsa-miR-98-5p: 5'GGGGGGTGAGGTAGTAAGTTGT3', 5’GTGCGTGTCGTGGAGTCG3’;

hsa-miR-151a-3p: 5'GGGCAACCTAGACTGAAGCTC3', 5’GTGCGTGTCGTGGAGTCG3’;

hsa-miR-191-5p: 5'GGCAACGGAATCCCAAAAG3', 5'GTGCGTGTCGTGGAGTCG3’.

**Upstream and downstream prediction of mir15a/mir16-1**

Prediction of transcription factors binding sites: An upstream region from 2000 bp from the transcriptional start site was defined as the promoter region of the DELU2 (pri-mir15a/mir16-1 host gene). Putative transcriptional factors (TFs) were identified using the Needleman-Wunsch algorithm and the TFs position weight matrix from JASPAR (<http://jaspar.genereg.net/>). Prediction of target genes: The putative target genes of mir15a/mir16-1 were analyzed using three databases online: Targetscan version 7.2, miRanda v3.3a and miRDB 6.0. To gain insight into the functions of the candidate miRNAs, functional enrichment analysis of putative target genes was performed using K[yoto Encyclopedia of Genes and Genomes](https://www.genome.jp/kegg/) (KEGG) database. To explore the key genes, putative target genes were analyzed in signal-net. These genes were connected in a network based on prior known protein-protein interaction and signaling pathways.

**Chromatin immunoprecipitation (ChIP) assays**

ChIP was performed as previously described^5^. Briefly, cells are treated with phenylephrine (PE) at dosage of 100nM for 24 hours. Cells were crosslinked with paraformaldehyde, quenched with glycine, lysed and then sonicated to an average of about 500bp. Sonicated chromatin was then immunoprecipitated by using 4ug of the anti-C/EBPβ (ab32358, abcam) or anti-HSF1(ab47369, abcam), anti-IgG (12-371B, Millipore) as the isotype control. The immunoprecipitate was collected by using protein A beads (Millipore, Temecula, CA, USA), and washed to remove non-specific DNA binding. Chromatin was eluted from beads, and crosslinks were removed overnight at 65°C. DNA was analyzed by real-time PCR. The promoter region of host gene DLEU2 contains 3 putative binding sites (BS) for C/EBPβ and 1 putative-binding site for HSF1 (Fig 3D). We designed 4 PCR amplicons to assay for the presence of these putative binding sites in CHIP. The following pair of primers was used:

C/EBPβ BS1: 5’ TTTTCTTGACGCTGAGGAGGAG 3’, 5’ TACCTAAAGTTAATGCCGTCACTGG 3’;

C/EBPβ BS2: 5’ TCTTTACCCGGACAACTTAACGG 3’, 5’ TAACAACTCTCGTCCCTCCTCC 3’;

C/EBPβ BS3: 5’ TTGACGCTGAAGCGTGAGGTG 3’, 5’ CTAGAGACCTCACCGGGACCCA 3’;

HSF1 BS: 5’ TTTTCTCGACCCGGTGATGG 3’, 5’ ACTGTTGCCACGACCCAC 3’.

**Luciferase reporter assays**

To construct promoter vectors, the wild-type pri-mir15a/mir16-1 promoter containing 2000 bp upstream of DLEU2 transcriptional start site were PCR-amplified employing the oligonucleotides. The C/EBPβ-binding sites in pri-mir15a/mir16-1 were subjected to site-directed mutagenesis to create the corresponding mutant constructs. Both wild-type and mutant promoter were inserted upstream of the Firefly luciferase reporter in the pGL3-basic vector. To construct miRNA 3’UTR luciferase reporter, the wild-type 3’UTR of INSR, IGF1R, AKT3 or SGK1, containing putative binding sites for mir15a/mir16-1, was PCR-amplified. The corresponding mutant constructs were created by mutating the seed regions of the mir15a/mir16-1-binding sites. Both wild-type and mutant 3’-UTRs were cloned downstream of the luciferase gene in the psiCHECK-2 luciferase vector. 293T cells were transfected with the appropriate plasmids in 24-well plates. 293T cells harvested and lysed for luciferase assay 48 hours after transfection. Luciferase assays were performed using a Dual-Luciferase Reporter Assay System (Promega, Madison, WI, USA) according to the supplier’s protocol. Firefly luciferase activity normalized to Renilla luciferase was used as an internal control. The experiments were performed in triplicate for each plasmid construct.

**SiRNA transfection**

Transfection was performed in CMs with short interfering RNAs (siRNAs) targeting C/EBPβ (sc-29862), INSR(sc-35673), IGF1Rα/β(sc-56638), AKT3(sc-38912), SGK1(sc-38914) and scrambled siRNAs (sc-37007) (Santa Cruz) at a final concentration of 100nM using Lipofectamine™ RNAiMAX reagent per manufacturer instructions.

**Generation of cardiac-specific mir15a/mir16-1 knockout mice**

B6.129S-Mirc30tm1.1Rdf/J mice, in which have *loxP* sites on either side of the *MIR15a/MIR16-1* cluster, are gift from Prof. Yiwei Chu’s Lab. Myh6-cre/Esr1 mice, in which the expression of Cre recombinase is under the control of the cardiac-specific Myh6 gene after tamoxifen induction, were purchase from Jackson Laboratory (Stock number 005650). Generation and genotyping of the B6.129S-Mirc30tm1.1Rdf/J mice^6^ and Myh6-cre/Esr1 mice^7^ has been described previously. To generate the cardiac-specific mir15a/mir16-1 knockout mice, we crossed B6.129S-Mirc30tm1.1Rdf/J mice with Myh6-cre mice. Mice were intraperitoneally treated with 0.1mg/g body weight of tamoxifen dissolved in corn oil (2mg/Kg) for 7 days at 8 week of age. In vivo deletion of the loxp-flanked the mir15a/mir16-1 allele was verified by PCR analysis.

**Animal models of cardiac hypertrophy**

All experiments were performed using 8-10 weeks old male mice. In the pressure-overload model, transverse aortic constriction (TAC) or sham operation. Briefly, after anaesthetization with pentobarbital, the aortic arch was ligated with a silk 6-0 suture; a 90% ligation was established using a 28-gauge needle. After the procedure, mice were closely monitored until full recovery. The sham-operated mice were underwent the same procedure leaving the suture untied.

**Transthoracic echocardiography**

Cardiac function was evaluated by the Vevo 770 high-resolution microimaging system (Visualsonic, Toronto, Canada) as previous reported^5^. Mice were lightly anesthetized with vaporized isoflurane (2.5% for induction, 1.0% for maintenance) in oxygen and lightly restrained in a supine position on a heated pad to maintain body temperature at 37° C. Heart rates were maintained between 500 and 600 beats per minute. The heart was viewed from the parasternal short-axis in 2D mode. The left ventricular wall thickness and chamber dimension were measured in M-mode.

**In vitro and in vivo delivery CHO-PEGA-miRNA complex**

Cholesterol (CHO)-terminated ethanolamine-aminated poly (glycidyl methacrylate) (CHO-PEGA) was used as transfer vector of miRNA. CHO-PEGA-miRNA complex was prepared as our previous report^8^. CHO-PEGA solution was dissolved in deionized water to achieve a nitrogen concentration of 10 mM and filtered. Polycation to miRNA ratios are expressed as molar ratios of nitrogen (N) in polymer to phosphate (P) in miRNA (or as N/P ratios). At the desired N/P ratios, equal volumes of CHO-PEGA and miRNA solutions were mixed, vortexed and incubated for 30 minutes at room temperature to produce CHO-PEGA-miRNA complex. Our previous study revealed that CHO-PEGA-miRNA complex at the N/P ratio of 10 was suitable for miRNA delivery into CMs^8^.

Cardiomyocytes (CMs) were isolated from one-week mouse pups by enzymatic digestion and cultured in CM-Medium as previous reported^9^. Isolated CMs were seeded in a 24-well plate (2$\times$10^5^ CMs per well). After 24 hours of culture, 20 μl of CHO-PEGA-miRNA complexs containing 1.0 μg miRNA were added into each well and kept for 4 hours. Then, the medium was replaced with fresh normal medium, followed by the follow-up experiments, including analysis of miRNA transfection efficiency, analysis of CM hypertrophy. For replenishment of mir15a/mir16-1 *in vivo*, we used CHO-PGEA to deliver mir15a/mir16-1 into the heart. The CHO-PEGA-miRNA complex were prepared in the N/P ratio of 10 and each injection dose of the complex containing 5 nM miRNA mimic was 100 μl. The CHO-PEGA-containing mir negative control (NC) mimic (10 nM/per mouse) or CHO-PEGA-containing mir15a/mir16-1 mimic (5/5 nM/per mouse) were administered to mice intravenously every three days from day 7 of post-surgery until the end of experiment.

**Fluorescence *in situ* hybridization (ISH)**

ISH analysis of mir15a and mir16 in cells was performed using the ViewRNA® Cell Plus Assay kit (Thermo Fisher Scientific, Waltham, MA, USA) and following the manufacturer’s instruction. Two probes were miR-16-5p Type4 Alexa Fluor 488 (gcgguuauaaaugcACGACGAu, Assay ID:VM4-10738) and miR-15a-5p Type1 Alexa Fluor 546 (guguuuGGUAAUAC-ACGACGAu, Assay ID:VM1-10230). We grew 50,000 cells in 24-well glass-bottom plates for 24 hours. After fixation and permeabilization, cells were labeled with primary antibodies α-actinin (A7732, Sigma), vimentin (ab45939, abcam), CD31 (ab9498, abcam) and Myh11 (ab53219, abcam), and then incubated with Alexa Fluor 633-conjugated secondary antibody (Jackson ImmunoResearch Laboratories, West Grove, PA). After the immunofluorescence, cells were incubated with the probe diluted 1:100 in probe set diluent for 2 hour 40 °C in a validated incubator, then with preamplifier mix at 40 °C for 1 hour, followed by amplifier mix at 40 °C for 1 hour, and finally label probe mix at 40 °C for 1 hour. Cells were washed and counterstained with DAPI and anti-fade reagent. Confocal microscopy was performed using a Leica ST5 laser scanning.

**qRT-PCR analysis of mRNA**

The 2 µg of total RNA from each heart sample was used to generate cDNA by using GoScript^TM^ reverse transcription system (Promega) according to the manufacturer’s instructions. qRT-PCR analysis was performed with SYBR Premix Ex Taq (Takara) by using 7500 Real-Time PCR systems (Applied Biosystems). Amplification was performed at 95°C for 4 minutes, 95°C for 45 seconds, and 60°C for 3 40 seconds for each step for 40 cycles. The expression of mRNA was normalized to the endogenous GAPDH. All primers are shown in the table below:

| Gene symbol | Human primers | Mouse primers |
| --- | --- | --- |
| STAT1 | 5’ATCAGGCTCAGTCGGGGAATA3’  5’TGGTCTCGTGTTCTCTGTTCT3’ | 5’ TCACAGTGGTTCGAGCTTCAG 3’  5’ CGAGACATCATAGGCAGCGTG 3’ |
| HSF1 | 5’ GCACATTCCATGCCCAAGTAT 3’  5’ GGCCTCTCGTCTATGCTCC 3’ | 5’ CCTGGCCCATACTCAGCTC 3’  5’ CTCTTGCTTGACACGGACC 3’ |
| STAT3 | 5’ ACCAGCAGTATAGCCGCTTC 3’  5’ GCCACAATCCGGGCAATCT 3’ | 5’ TGTGACACCATTCATTGATGC 3’  5’ AGCTTCTGGTTTCAGCTCCTC 3’ |
| IRF1 | 5’ ATGCCCATCACTCGGATGC 3’  5’ CCCTGCTTTGTATCGGCCTG 3’ | 5’ ATGCCAATCACTCGAATGCG 3’  5’ CCTGCTTTGTATCGGCCTGT 3’ |
| C/EBPβ | 5’ CTTCAGCCCGTACCTGGAG 3’  5’ GGAGAGGAAGTCGTGGTGC 3’ | 5’ CAAGAACAGCAACGAGTACCG 3’  5’ GTCACTGGTCAACTCCAGCAC 3’ |
| DDIT3 | 5’ CAGGAGGTCCTGTCCTCAGA 3’  5’ CTGTCAGCCAAGCTAGGGAC 3’ | 5’ GCCAGAATAACAGCCGGAACC 3’  5’ ATGCACTTCCTTCTGGAACACT 3’ |
| ELK1 | 5’ TCCCTGCTTCCTACGCATACA 3’  5’ GCTGCCACTGGATGGAAACT 3’ | 5’ TTGTGTCCTACCCAGAGGTTG 3’  5’ GCTATGGCCGAGGTTACAGA 3’ |
| EP300 | 5’ ATGAGTCCCCAAGCTCAGCA 3’  5’ GTTGCATCATCTGCCGTCT 3’ | 5’ CACAATCCCCCGTGATGCTA 3’  5’ GAAATGATACCTGTTCTGGTAACTG 3’ |
| EPAS1 | 5’ CTGAGGCGGCCGTACAATC 3’  5’ GGGCTGAGCTGACCATACAG 3’ | 5’ GTGACCCAAGACGGTGACAT 3’  5’ CACGGATCTCCTCATGGTCG 3’ |
| NFKB1 | 5’ GGGCAGGAAGAGGAGGTTTC 3’  5’ GAAGGGCAGGGGAAGCTG 3’ | 5’ GGCATCCACCATGGAAGACA 3’  5’ AGCTGCAGAGCCTTCTCAAG 3’ |
| NFYA | 5’CAGTGGAGGCCAGCTAATCAC3’  5’ CCAGGTGGGACCAACTGTATT 3’ | 5’ AGCCGTTAATGGTGCAAGTCA 3’  5’ ACCCTGAATCTGGATCTGTCC 3’ |
| NKX2.5 | 5’ CCAAGGACCCTAGAGCCGAA 3’  5’ CCAGGTGGGACCAACTGTATT 3’ | 5’ TGGGACCTTTCTCCGATCCA 3’  5’ TCCCGGTCCTAGTGTGGAAT 3’ |
| RORA | 5’ CTTGCCGTAGGGATGTCTCG 3’  5’ GAAGTTCCGTCAGCCCGTT 3’ | 5’ GCTGACCCAGGACACGG 3’  5’ ACTGAGATACCTCGGCTGGA 3’ |
| RELA | 5’ CCGGGATGGCTTCTATGAGG 3’  5’ GGTTGTTGTTGGTCTGGATGC 3’ | 5’ GCCTCTGGCGAATGGCTTTA 3’  5’ TCCACATAAGGCCCAGAAGC 3’ |
| RREB1 | 5’ CAGTAACACGTCCCCAGGAG 3’  5’ CCTGGAACACACAGTCGGAG 3’ | 5’ GCACATCCAGAAACGCCATT 3’  5’ TGGAGCACAGGATTGCAACA 3’ |
| SREBF1 | 5’ TTGCTGCTTCTAACCTGGCA 3’  5’ GATAGCATCTCCTGCGCACT 3’ | 5’ CAGACTCACTGCTGCTGACA 3’  5’ GATGGTCCCTCCACTCACCA 3’ |
| SRF | 5’ AGCGATAGCGGCACTAGCAG 3’  5’ CTTGGGTCGGTAACATGGCG 3’ | 5’ AGCGATAGCGGCACTAGCAG 3’  5’ CTTGGGTCGGTAACATGGCG 3’ |
| XBP1 | 5’ GTTAAGACAGCGCTTGGGGA 3’  5’ TGCACGTAGTCTGAGTGCTG 3’ | 5’ GTTAAGACAGCGCTTGGGGA 3’  5’ TGCACGTAGTCTGAGTGCTG 3’ |
| ZEB1 | 5’ ACAAGCGAGAGGATCATGGC 3’  5’ CTGCTTTCTGCGCTTACACC 3’ | 5’ ACAAGCGAGAGGATCATGGC 3’  5’ CTGCTTTCTGCGCTTACACC 3’ |
| ANP | 5’ CAACGCAGACCTGATGGATTT 3’  5’ AGCCCCCGCTTCTTCATTC 3’ | 5’ GTACCCAGGCATTGCTGACA 3’  5’ GCTGGAAGGTAGACAGCGAA 3’ |
| GAPDH | 5’ CTGGGCTACACTGAGCACC 3’  5’ AAGTGGTCGTTGAGGGCAATG 3’ | 5’ AATGCATCCTGCACCACC 3’  5’ ATGCCAGTGAGCTTCCCG 3’ |

**Western blot analysis**

Total proteins from ventricular tissue or CMs were extracted by SDS lysis buffer containing a protease inhibitor cocktail and phosphatase inhibitors (Roche, Mannheim, Germany). After blocking nonspecific binding with 5% bovine serum albumin (Biotopped, Beijing, China), membranes were then incubated overnight at 4°C with primary antibodies against C/EBPβ (ab32358, abcam), INSR-β subunit (YT2361, Immunoway), IGF1R (ab39398, abcam), AKT3 (#3788, CST), p-AKT (#13038, CST), SGK1 (ab59337, abcam), p-SGK1 (ab55281, abcam), IRS1 (ab52167, abcam), p-IRS1(#2382, CST), p-mTOR (#5536, CST), mTOR (#2972, CST), p-ERK1/2 (#4370, CST), ERK1/2 (#4695, CST), GAPDH (#5174, CST), β-tubulin (#2148, CST), followed by infrared Dye 800-conjugated secondary antibodies (Rockland Immunochemicals, Gilbertsville, PA). Signals were detected using the LiCor/Odyssey infrared image system (LI-COR; Biosciences, Lincoln, NE, USA).

**Histopathology analysis**

Histopathology analysis was performed as previous reported^5^. Briefly, heart tissues were fixed, embedded, and sectioned into 5μm thick slices. Sections were stained with Masson’s trichrome (collagen, blue; cytoplasm, red/pink) for collagen deposition analysis. To assess the degree of cardiac fibrosis, we calculated the ratio of tissue area stained blue (collagen) to the left ventricle area (10-12 randomly chosen sections per heart). Sections were stained with wheat germ agglutinin (WGA) to assess cellular hypertrophy. Immunohistochemistry images were captured by a Nikon Eclipse TE2000-S microscope (Nikon, Japan), and analyzed by a person blinded to treatment using Image Pro Plus 3 (Nikon). Immunofluorescence images were captured with Leica ST5 laser scanning confocal microscope.

**Proteomics analysis with tandem mass tags (TMT) labeling**

The heart tissues were lysed, extracted and digested as previous reported^10^. For relative quantification of the global proteome by liquid chromatography tandem mass spectrometry (LC-MS/MS), 100μg peptide per sample was labled with 10-plexing TMT (Thermo scientific) following the manufacture instruction. The labeled peptides were combined and fractionated using high-pH reversed phase chromatography into 10 fractions. Each fraction was loaded on onto Q Exactive Plus (Thermo Fisher Scientific) coupled to Easy nLC (Thermo Fisher Scientific) for 90 min (Thermo Fisher Scientific, Acclaim PepMap RSLC 50um X 15cm, nano viper, P/N164943). The gradient is: 6% buffer B for 5 min, 6-28% buffer B for 63 min, 28-38% buffer B for 10 min, 38-100% buffer B for 7 min, hold in 100% buffer B for 5 min. The mass spectrometer was operated in positive ion mode. MS data was acquired using a data-dependent top10 method dynamically choosing the most abundant precursor ions from the survey scan (350–1800 m/z) for HCD fragmentation. Automatic gain control (AGC) target was set to 3$\times$10^6^, and maximum inject time to 45 ms. Survey scans were acquired at a resolution of 70,000 at m/z 200 and resolution for HCD spectra was set to 17,500 at m/z 200, and isolation width was 2 m/z. Normalized collision energy was 30 eV. MS/MS spectra were searched against Uniprot MusMusculus database (Version 20170123, 84433 entries) using MASCOT engine (Matrix Science, London, UK; version 2.6) embedded into Proteome Discoverer 2.1. We used the Student’s T test to filter the differentially expressed proteins (DEPs). DEPs were defined by a fold change $\geq1.$2 and a false discovery rate (FDR)-corrected p<0.05. Pathway enrichment analysis was performed using KEGG database. Correlation of fold-change in protein expression between mir15a/mir16-1-TAC vs mir NC-TAC (y-axis) and mirNC-TAC vs Sham (x-axis) was used with Pearson’s correlation analysis as previous reported^11^.

**Electrophoretic mobility shift assay (EMSA)**

The 293T cells were infected with or without C/EBPβ expression vectors. At 48 hour post-infection, nuclear proteins were extracted using a commercial extraction kit (Pierce Biotechnology), and lysate concentrations quantified. Oligonucleotides were constructed based on the C/EBPβ binding site on the DLEU2 promoter. Biotin end-labeled probe was prepared: WT C/EBPβ-F- TCAATTCTTACTCCACAG, WT C/EBPβ-R- CTGTGGAGTAAGAATTGA; Mut C/EBPβ-F- -TCAATTCAAGGAAGGCAG, Mut C/EBPβ-R-CTGCCTTCCTTGAATTGA. Reactions were carried out in accordance with manufacturer's protocol for LightShift Chemiluminescent EMSA kit (Pierce Cat# 20148). For each binding reaction, 2 μg nuclear extract was used. Reaction mixtures were subjected to PAGE on 6% gels in 0.5× TBE buffer at 100 V. DNA was transferred to positively charged nylon membranes, crosslinked for 10 min at 254 nm using a UV transilluminator, and then probed with HRP-conjugated streptavidin primary antibody (1:300 v/v). EMSA supershift analysis was performed by adding 2 μg of the of C/EBPβ antibody (Abcam, ab32358) to the binding reactions for 15 min at room temperature prior to the addition of the DNA probe.

**Statistical analysis**

Receiver operating characteristic (ROC) curves were analyzed to assess discrimination ability of single miRNAs. Spearman’s correlation analysis was performed to determine the relationships between hypertrophic severity and miRNAs. Cox regression models, both unadjusted and adjusted, were used to calculate the hazard ratios associated with outcome by the serum mir15a and mir16-1 at admission. Cumulative incidence of LVH was derived by the Kaplan–Meier method. Group-wise comparisons were based on the log-rank test. Statistical analyses were performed using R version 3.3.3 (R Foundation for Statistical Computing, Vienna, Austria) and SPSS version 23.0 (IBM Corp.). A value of p<0.05 was considered significant.

**References**

1. Authors/Task Force m*, et al.* 2014 ESC Guidelines on diagnosis and management of hypertrophic cardiomyopathy: the Task Force for the Diagnosis and Management of Hypertrophic Cardiomyopathy of the European Society of Cardiology (ESC). *Eur Heart J* **35**, 2733-2779 (2014).

2. Devereux RB, *et al*. Echocardiographic assessment of left ventricular hypertrophy: comparison to necropsy findings. *Am J Cardiol* **57**, 450-458 (1986).

3. de Simone G*, et al.* Cardiovascular risk in relation to a new classification of hypertensive left ventricular geometric abnormalities. *J Hypertens* **33**, 745-754; discussion 754 (2015).

4. Livak KJ, Schmittgen TD. Analysis of relative gene expression data using real-time quantitative PCR and the 2(-Delta Delta C(T)) Method. *Methods* **25**, 402-408 (2001).

5. Li Y*, et al.* Cardiac Fibroblast-Specific Activating Transcription Factor 3 Protects Against Heart Failure by Suppressing MAP2K3-p38 Signaling. *Circulation* **135**, 2041-2057 (2017).

6. Klein U*, et al.* The DLEU2/miR-15a/16-1 cluster controls B cell proliferation and its deletion leads to chronic lymphocytic leukemia. *Cancer Cell* **17**, 28-40 (2010).

7. Tian Y*, et al.* A microRNA-Hippo pathway that promotes cardiomyocyte proliferation and cardiac regeneration in mice. *Sci Transl Med* **7**, 279ra238 (2015).

8. Zhi Y, Xu C, Sui D, Du J, Xu FJ, Li Y. Effective Delivery of Hypertrophic miRNA Inhibitor by Cholesterol-Containing Nanocarriers for Preventing Pressure Overload Induced Cardiac Hypertrophy. *Adv Sci (Weinh)* **6**, 1900023 (2019).

9. Li Y*, et al.* S100a8/a9 Signaling Causes Mitochondrial Dysfunction and Cardiomyocyte Death in Response to Ischemic/Reperfusion Injury. *Circulation* **140**, 751-764 (2019).

10. Wiśniewski JR, Zougman A, Nagaraj N, Mann M. Universal sample preparation method for proteome analysis. *Nature Methods* **6**, 359-362 (2009).

11. Hu H*, et al.* Mutational Landscape of Secondary Glioblastoma Guides MET-Targeted Trial in Brain Tumor. *Cell* **175**, 1665-1678 e1618 (2018).


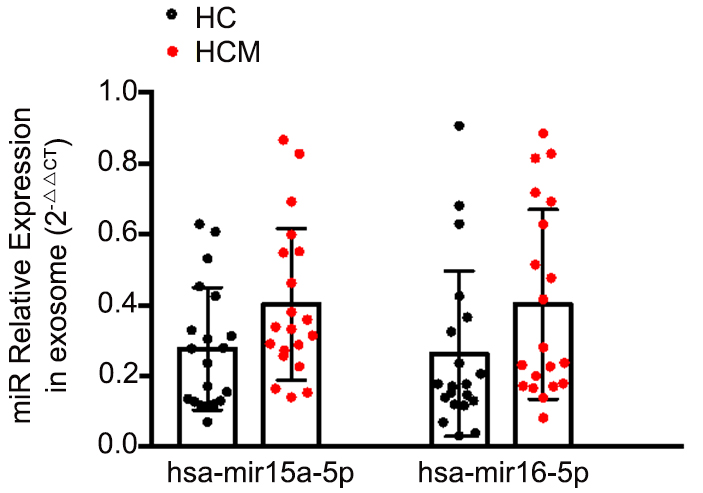


**Supplemental Figure 1. Expression of mir15a/mir16-1 in plasma-derived exosomes.**

**A** qRT-PCR showing mir15a-5p and mir16-5p expression in exosomes isolated from plasma of HCM patients and HC (n=20).

Statistical significance was determined by the two-sided t-test.


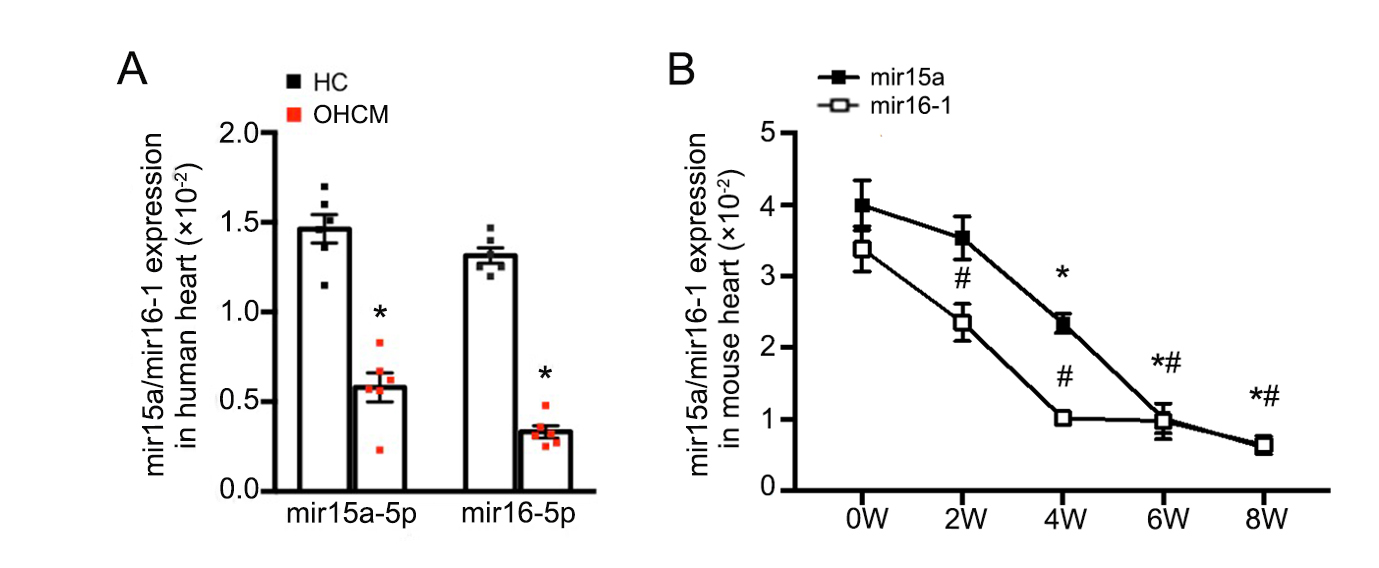


**Supplemental Figure 2. Expression of mir15a/mir16-1 in cardiac tissue.**

**A** qRT-PCR showing mir15a-5p and mir16-5p expression in human samples from representative normal control hearts and hypertrophic hearts from patients from obstructive HCM (n=6/per group). *p<0.05 vs HC.

**B** qRT-PCR showing mir15a and mir16-1 expression in heart samples from mice subjected to TAC (n=6 per group). *p<0.05 vs mir15a before TAC; ^#^p<0.05 vs mir16-1 before TAC.

Statistical significance was determined by the two-sided t-test (A) or by repeated measures analysis (B).

**
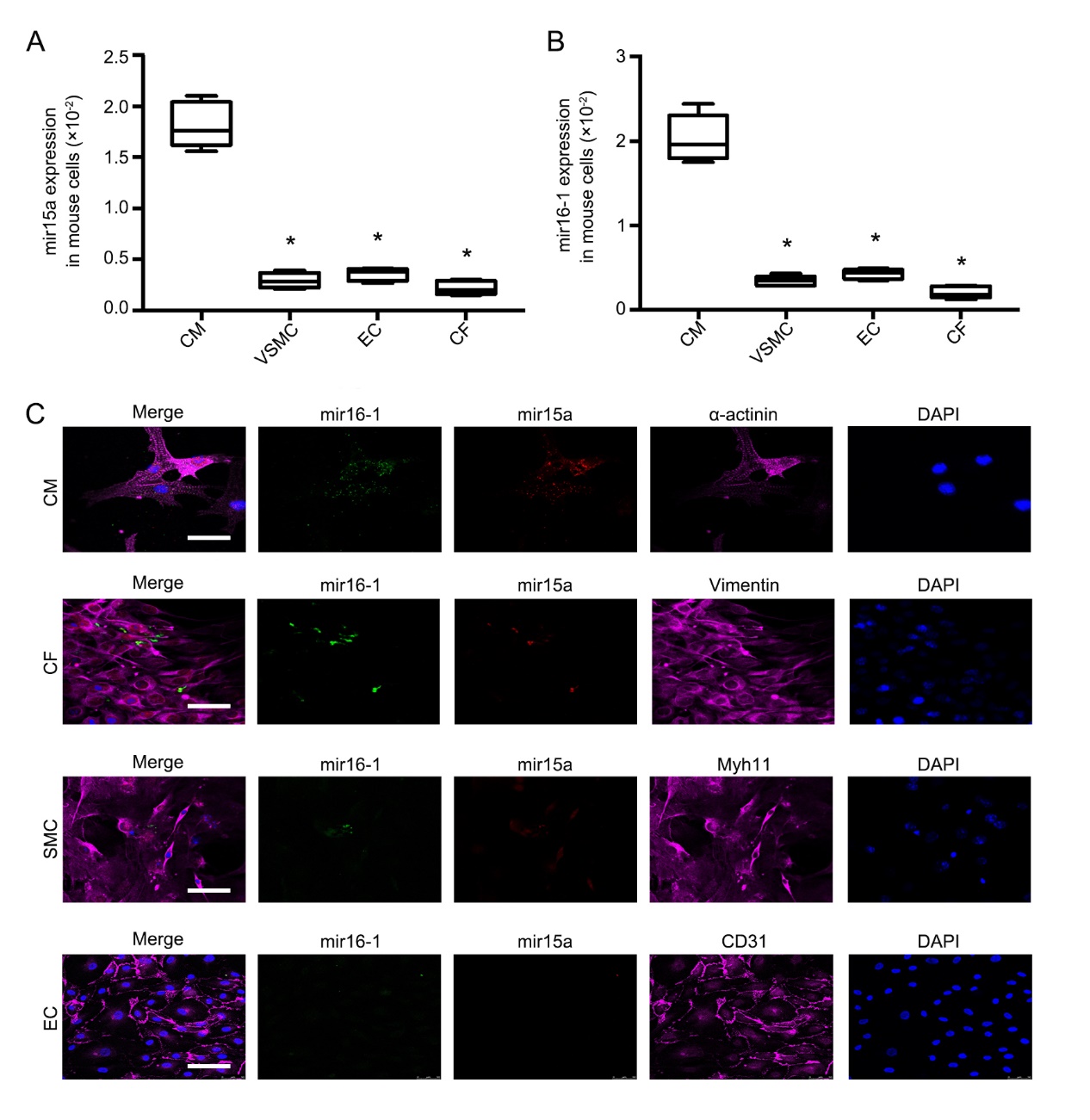
**

**Supplemental y Figure 3. Expression of mir15a/mir16-1 is enriched in cardiomyocytes.**

**A, B** qRT-PCR showing mir15a and mir16-1 expression in isolated neonatal cardiomyocytes (CMs), cardiac fibroblasts (CFs), aortic endothelial cells (ECs) and aortic vascular smooth muscle cells (VSMCs) from wild type (WT) mice (n=5). *p<0.05 vs. CMs.

**C** *In situ* hybridization of mir15a and mir16-1in CMs, CFs, ECs and VSMCs from WT mice. Scale bar=25μm.

Statistical significance was determined by 1-way ANOVA Tukey’s post hoc test (A, B).


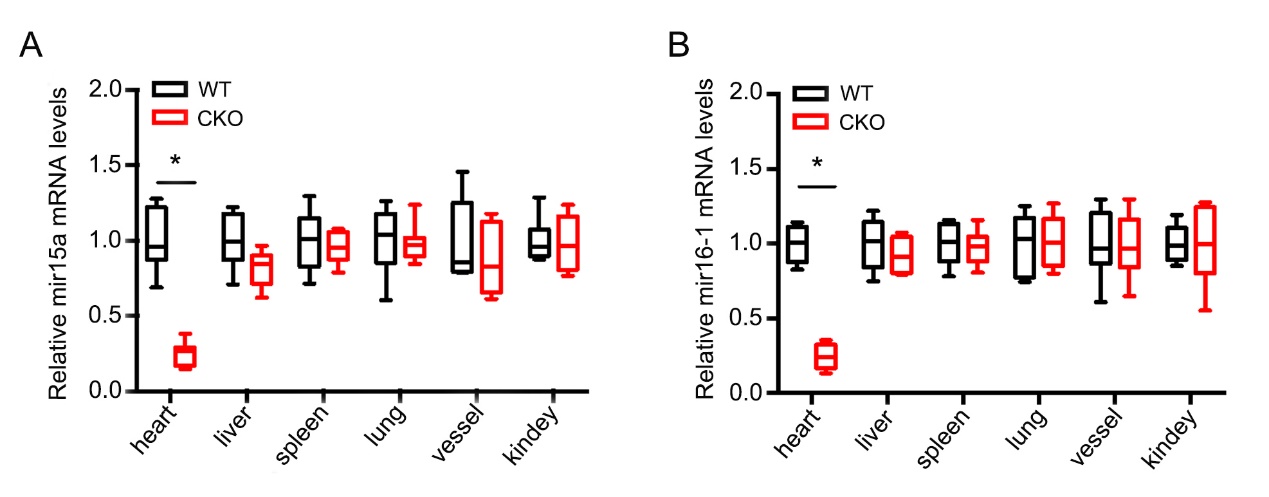


**Supplemental Figure 4. Identification of cardiomyocyte-specific mir15a/mir16-1 deficient.**

**A, B** Cardiomyocyte-specific mir15a/mir16-1 knockout (CKO) and wild-type (WT) control mice were subjected to tamoxifen for 7 days, then recovered for another 7 day. qRT-PCR showing expression of mir15a **(A)** and mir16-1 **(B)** in heart, liver, spleen, vessel and kidney from cardiomyocyte-specific mir15a/mir16-1 knockout (CKO) and wild-type (WT) control mice after tamoxifen administration. (n=8/per group). *p<0.05 vs WT mice.

Statistical significance was determined by the two-sided t-test (A, B).


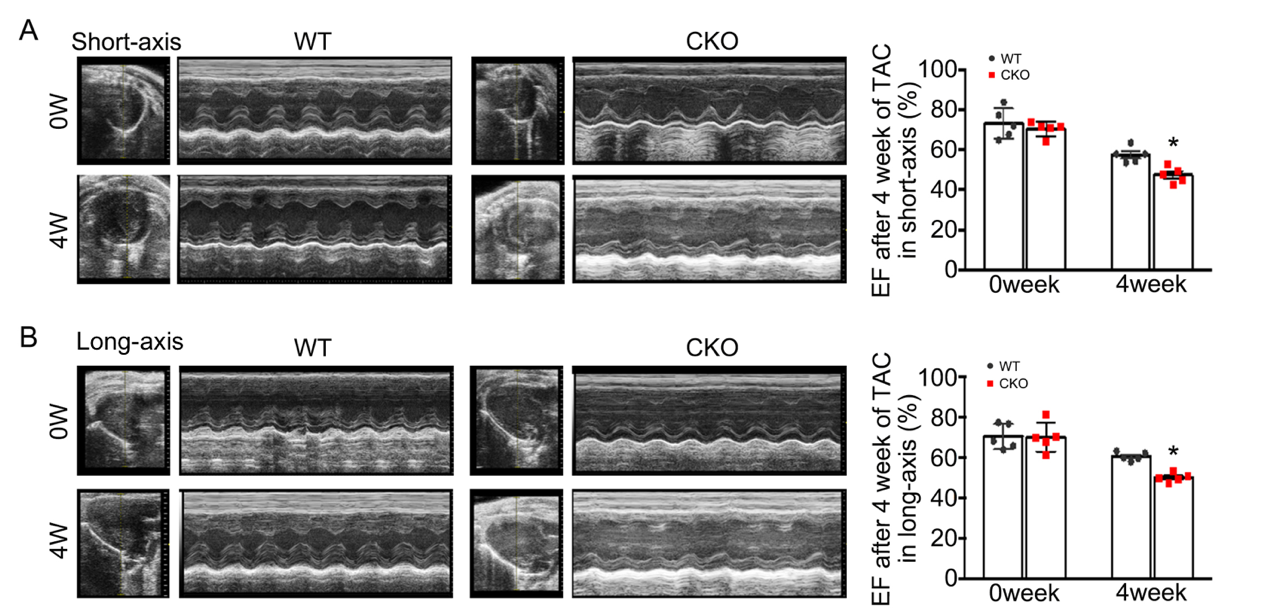


**Supplemental Figure 5. Cardiac function by measured echocardiography.**

M-mode echocardiographic imaging of heart and analysis of ejection fraction (EF) at 0 and 4 weeks of TAC in short-axis (**A**) and long-axis (**B**) (n=5 per group). *p<0.05 vs. WT mice.

Statistical significance was determined by the two-sided t-test (A, B).


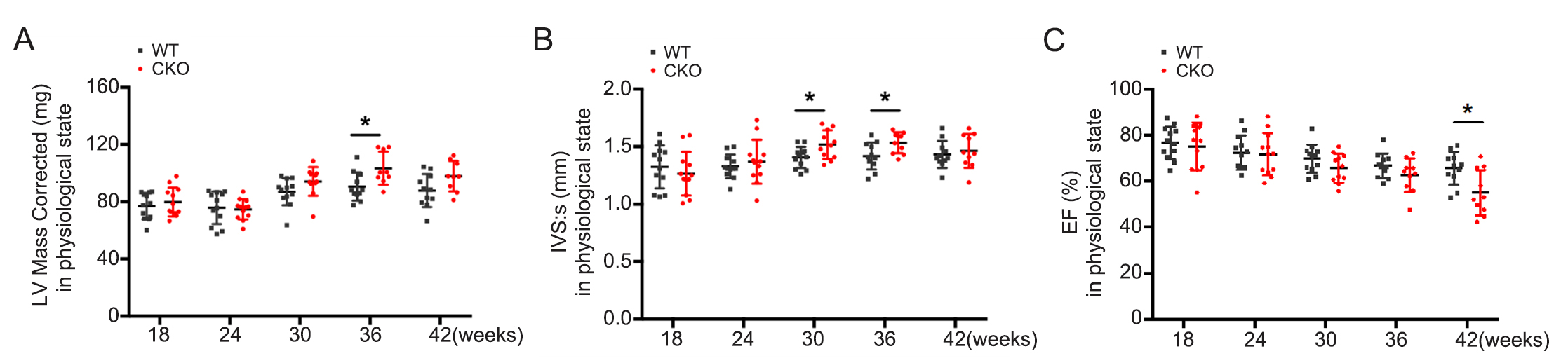


**Supplemental Figure 6. Echocardiographic assessment of cardiomyocyte-specific mir15a/mir16-1 knockout mice with aging.**

**A-C**. Analysis of and LV mass corrected (**A**), interventricular septum thickness (IVS):s (**B**) and ejection fraction (EF)% (**C**) of hearts of WT and CKO during the physiological state (n=10-12 per group). *p<0.05 vs. WT mice.


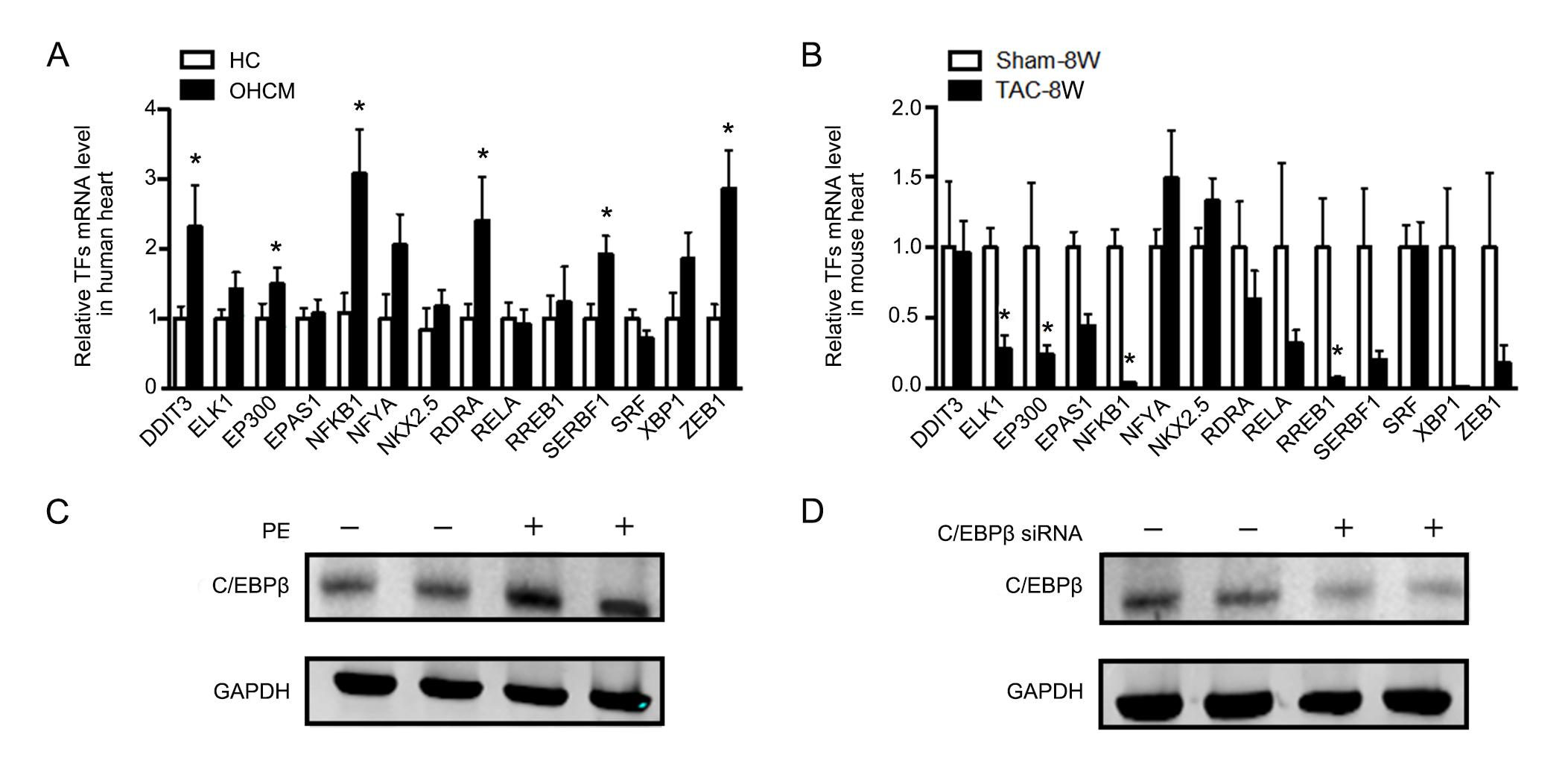


**Supplemental Figure 7. Candidate TFs expression in hearts.**

**A** qRT-PCR showing expression of transcription factors (TFs) in human samples from representative normal control hearts and hypertrophic hearts (n=6/per group). *p<0.05 vs HC.

**B** qRT-PCR showing expression of TFs in hearts samples from mice subjected to TAC (n=6 per group). *p<0.05 vs sham operation.

**C** Western blot analysis of C/EBPβ proteins levels in CMs stimulated with PE (n=2 experiments with 2 well replicates).

**D** Western blot of C/EBPβ proteins levels in CMs transfected by control-siRNA or CEBPβ-siRNA (n=2 experiments with 2 well replicates).

Statistical significance was determined by the two-sided t-test (A, B).


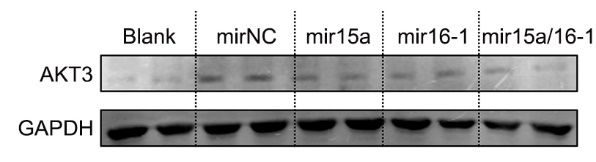


**Supplemental Figure 8. Effect of mir15a/mir16-1 on AKT3 expression in human cardiomyocytes-like cells.**

Western blot analysis of AKT3 expression in human cardiomyocytes-like AC16 cells untreated (blank) or treated with mimic-mirNC, mimic-mir15a, mimic-mir16-1, or mimic-mir15a plus mimic-mir16-1. GAPDH levels served as loading control.

**
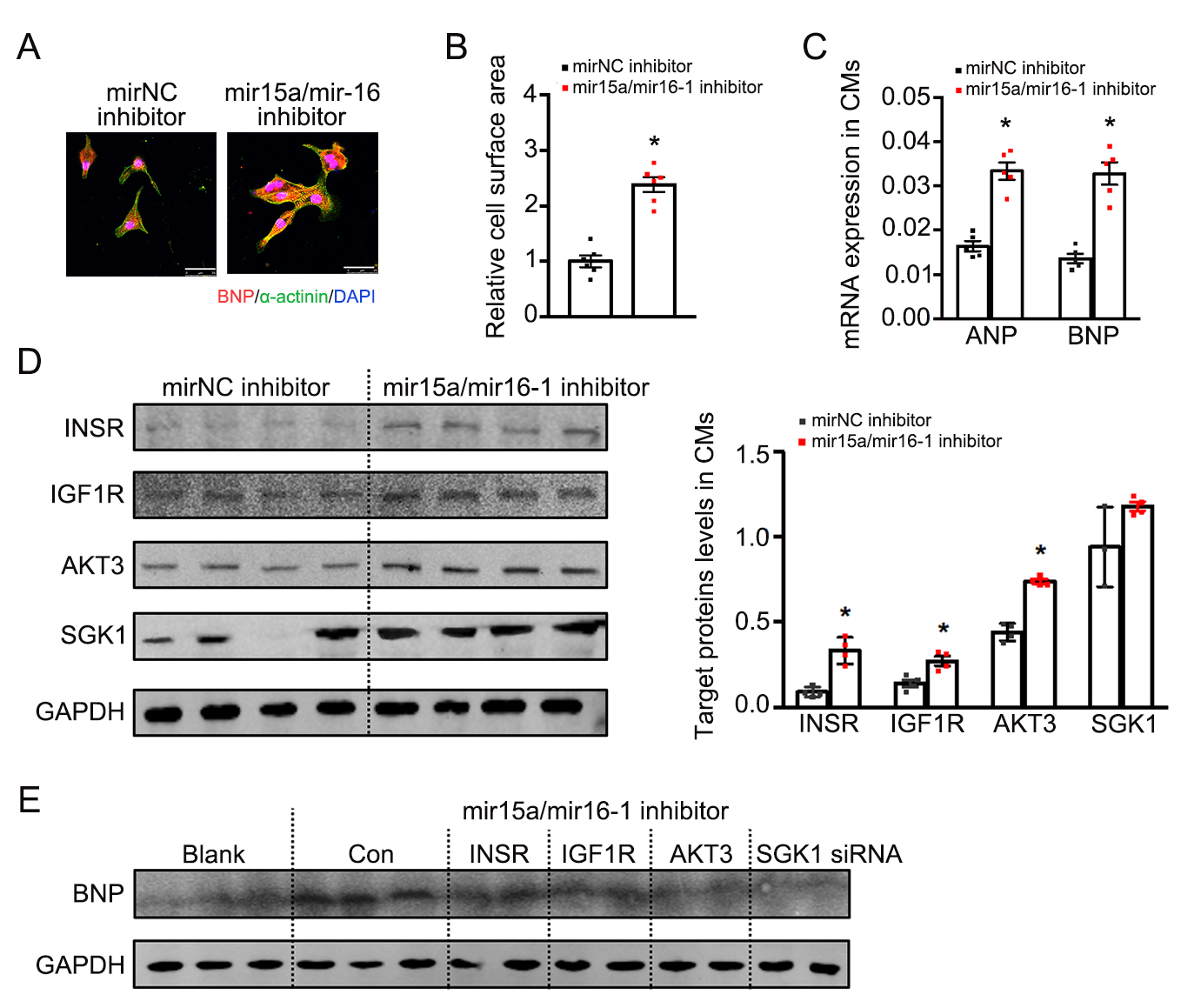
**

**Supplemental Figure 9. Effect of mir15a/mir16-1 inhibitor on target genes and hypertrophic response *in vitro*.**

**A-C** CMs were treated with mir15a/mir16-1 inhibitor (50nM) and mirNC inhibitor (50nM) for 48 hours. Hypertrophy was assessed by morphological change (**A**, bar=25um), cell surface area measurement (**B**), and mRNA expression of ANP and BNP (**C**) (n=5 experiments with 2 well replicates). *p<0.05 vs. mirNC inhibitor.

**D** Western blot analysis of proteins levels (INSR, IGF1R, AKT3, SGK1) in CMs transfected with mirNC or mir15a/mir16-1 inhibitor treatment (50nM) in the presence of PE (n=2 experiments with 2 well replicates). *p<0.05 vs. mirNC inhibitor.

**E** CMs were transfected with control, INSR, IGF1R, AKT3, or SGK1-siRNA for 24 hours. CMs were then treated with mir15a/mir16-1 inhibitor and PE for another 48 hours. Hypertrophy was assessed by the levels of BNP proteins (n=2 experiments with 2 well replicates).

Statistical significance was determined by the Mann–Whitney U test (A).

**
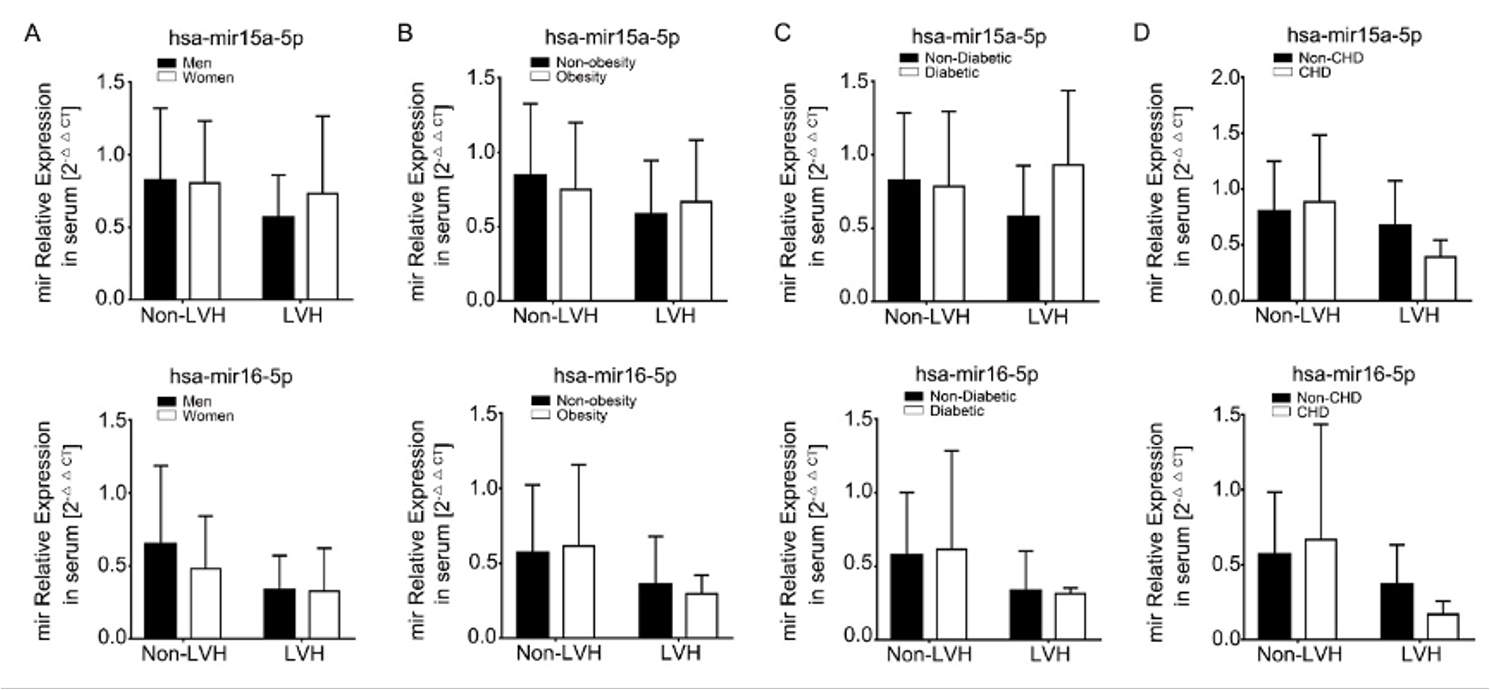
**

**Supplemental Figure 10. Expression of mir15a/mir16-1 in the subgroup of hypertension patients.**

**A-D** Serum mir15a and mir16-1 expression at admission in the hypertension patients with or without incident LVH among the different subgroup, including gender **(A)**, obesity **(B)**, diabetes **(C)** and coronary heart disease **(D)**. Coronary heart disease=CHD.

All statistical significance was determined by the two-sided t-test.


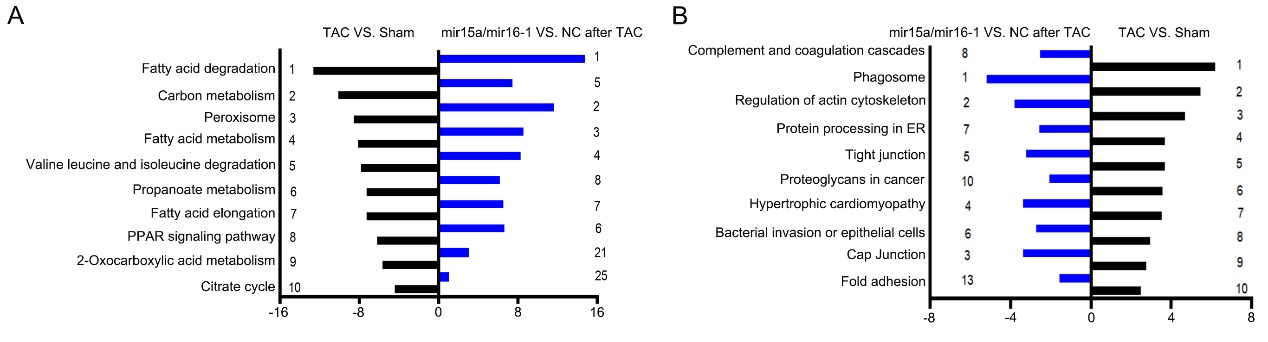


**Supplemental Figure 11. KEGG analysis.**

Bar showing KEGG terms that were significantly pathway between Sham-mir NC vs TAC-mir NC and TAC-mir NC vs. TAC-mir15a/mir16-1. The color shows the significance of upregulated (blue) and downregulated (red) pathway.

**
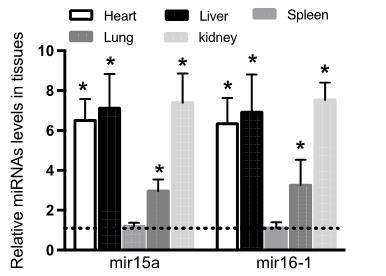
**

**Supplemental Figure 12. Effect of mir15a/mir16-1 overexpression in internal organs.**

qRT-PCR revealing cardiac mir15a and mir16-1 expression in mice subjected to CHO-PEGA-mirNC or mir15a/mir16-1 treatment for 4 weeks (n=8 per group). The miRNAs levels were presented with the ratio of CHO-PEGA-mir15a/mir16-1 to CHO-PEGA-mirNC in heart, liver, spleen, lung and kidney (n=4 per groups). *p<0.05 vs. CHO-PEGA-mir NC-TAC.

Statistical significance was determined by the two-sided t-test.

**Supplemental Table 1. Clinical characteristics of participators in the discovery phase.**

|  | **HC**  (n=9) | **HCM**  (n=15) | **NOHCM**  (n=7) | **OHCM**  (n=8) | **P values**  (HC vs HCM) | **P values**  (NOHCM vs OHCM) |
| --- | --- | --- | --- | --- | --- | --- |
| **Demographics** |  |  |  |  |  |  |
| Age, y | 42.1 ± 4.6 | 40.9 ± 10.4 | 38.4±12.0 | 43.0 ± 9.1 | **0.009** | 0.417 |
| Male, n (%) | 4 (44.4) | 6 (40.0) | 3 (42.9) | 3 (37.5) | 1.000 | 1.000 |
| **Comorbidity** |  |  |  |  |  |  |
| Syncopes, n (%) | 0 (0) | 3 (20.0) | 0 (0) | 3 (37.5) | 0.266 | 0.200 |
| Positive family history, n (%) | 0 (0) | 7 (46.7) | 3 (42.9) | 4 (50.0) | **0.022** | 1.000 |
| Dyspnoea, n (%) | 0 (0) | 5 (33.3) | 1 (14.3) | 4 (50.0) | 0.118 | 0.282 |
| Angina pectoris, n (%) | 0 (0) | 1 (6.7) | 1 (14.3) | 0 (0) | 1.000 | 0.467 |
| Palpitations, n (%) | 0 (0) | 2 (13.3) | 0 (0) | 2 (25.0) | 0.511 | 0.467 |
| Hypertension, n (%) | 0 (0) | 3 (20.0) | 2 (28.6) | 1 (12.5) | 0.266 | 0.569 |
| Atrial fibrillation, n (%) | 0 (0) | 0 (0) | 0 (0) | 0 (0) | NA | NA |
| Diabetes mellitus, n (%) | 0 (0) | 1 (6.7) | 0 (0) | 1 (12.5) | 1.000 | 1.000 |
| Hyperlipemia, n (%) | 0 (0) | 0 (0) | 0 (0) | 0 (0) | NA | NA |
| **Medication treatment** |  |  |  |  |  |  |
| *β*-blockers, n (%) | 0 (0) | 1 (6.7) | 1 (14.3) | 0 (0) | 1.000 | 0.467 |
| ACEI/ARB, n (%) | 0 (0) | 2 (13.3) | 2 (28.6) | 0 (0) | 0.511 | 0.200 |
| Calcium antagonists, n (%) | 0 (0) | 6 (40.0) | 2 (28.6) | 4 (50.0) | 0.052 | 0.608 |
| **Echocardioraphy** |  |  |  |  |  |  |
| IVS, mm | 9.3 ± 1.0 | 19.8 ± 4.9 | 16.1 ± 1.9 | 23.0 ± 4.4 | **<0.001** | **0.003** |
| LVPW, mm | 8.8 ± 0.7 | 11.0 ± 2.1 | 11.0 ± 2.1 | 11.0 ± 2.3 | **0.008** | 1.000 |
| IVS/LVPW | 1.1 ± 0.1 | 1.8 ± 0.5 | 1.5 ± 0.3 | 2.1 ± 0.4 | **<0.001** | **0.008** |
| LVEDD, mm | 47.9 ± 1.8 | 43.7 ± 5.6 | 42.9 ± 3.7 | 44.5 ± 7.1 | **0.016** | 0.723 |
| LVESD, mm | 36.9 ± 1.9 | 24.5 ± 9.9 | 27.9 ± 4.0 | 21.6 ± 12.7 | **<0.001** | 0.560 |
| LVOT PG, mmHg | 3.6 ± 1.1 | 47.6 ± 48.7 | 6.4 ± 1.6 | 83.6 ± 39.5 | **0.004** | **<0.001** |
| LVEF, % | 65.9 ± 3.4 | 67.8 ± 6.2 | 66.1 ± 2.5 | 69.3 ± 8.1 | 0.405 | 0.351 |
| LVFS, % | 37.6 ± 4.8 | 35.5 ± 7.0 | 31.4 ± 5.1 | 39.1 ± 6.7 | 0.411 | **0.028** |
| SAM, n (%) | 0 (0) | 7 (46.7) | 0 (0) | 7 (87.5) | **0.022** | **0.001** |

Values are mean(±SD) or n (%).
HC=healthy control; NOHCM=non-obstructive hypertrophic cardiomyopathy; OHCM= obstructive hypertrophic cardiomyopathy; ACEI=angiotensin-converting enzyme inhibitor; ARB=angiotensin receptor blocker; IVS=interventricular septum; LVPW=left ventricular posterior wall; LVEDD=left ventricular end-diastolic diameter; LVESD=left ventricular end-systolic diameter; LVOT PG=left ventricular outflow tract pressure gradient; LVEF=left ventricular ejection fraction; LVFS=left ventricular fractional shortening; SAM=systolic anterior motion.

**Supplemental Table 2. Clinical characteristics of participators in the validation phase.**

|  | **HC**  (n=30) | **HCM**  (n=78) | **NOHCM**  (n=25) | **OHCM**  (n=53) | **P values**  (HC  vs HCM) | **P values**  (NOHCM  vs OHCM) |
| --- | --- | --- | --- | --- | --- | --- |
| **Demographics** |  |  |  |  |  |  |
| Age, y | 47.6 ± 6.7 | 51.0 ± 12.6 | 50.4±11.6 | 51.3 ± 13.1 | 0.074 | 0.660 |
| Male, n (%) | 10 (33.3) | 26 (33.3) | 8 (32.0) | 18 (34.0) | 1.000 | 0.864 |
| **Comorbidity** |  |  |  |  |  |  |
| Syncopes, n (%) | 0 (0) | 29 (37.2) | 2 (8.0) | 27 (50.9) | **<0.001** | **<0.001** |
| Positive family history, n (%) | 0 (0) | 12 (15.4) | 2 (8.0) | 10 (18.9) | 0.053 | 0.365 |
| Dyspnoea, n (%) | 0 (0) | 49 (62.8) | 8 (32.0) | 41 (77.4) | **<0.001** | **<0.001** |
| Angina pectoris, n (%) | 0 (0) | 15 (19.2) | 5 (20.0) | 10 (18.9) | **0.023** | 1.000 |
| Palpitations, n (%) | 0 (0) | 25 (32.1) | 7 (28.0) | 18 (34.0) | **<0.001** | 0.598 |
| Hypertension, n (%) | 0 (0) | 25 (32.1) | 8 (32.0) | 17 (32.1) | **<0.001** | 0.995 |
| Atrial fibrillation, n (%) | 0 (0) | 7 (9.0) | 3 (12.0) | 4 (7.5) | 0.208 | 0.828 |
| Diabetes mellitus, n (%) | 0 (0) | 4 (5.1) | 2 (8.0) | 2 (3.8) | 0.487 | 0.811 |
| Hyperlipemia, n (%) | 0 (0) | 13 (16.7) | 7 (28.0) | 6 (11.3) | **0.040** | 0.129 |
| **Medication treatment** |  |  |  |  |  |  |
| *β*-blockers, n (%) | 0 (0) | 30 (38.5) | 11 (44.0) | 19 (35.8) | **<0.001** | 0.490 |
| ACEI/ARB, n (%) | 0 (0) | 20 (25.6) | 7 (28.0) | 13 (24.5) | **0.002** | 0.743 |
| Calcium antagonists, n (%) | 0 (0) | 42 (53.8) | 8 (32.0) | 34 (64.2) | **<0.001** | **0.008** |
| **Echocardioraphy** |  |  |  |  |  |  |
| IVS, mm | 9.4 ± 0.9 | 20.5 ± 4.7 | 15.8 ± 2.0 | 22.7 ± 3.9 | **<0.001** | **<0.001** |
| LVPW, mm | 8.8 ± 0.6 | 12.5 ± 3.2 | 11.5 ± 3.0 | 13.0 ± 3.3 | **<0.001** | **0.024** |
| IVS/LVPW | 1.1 ± 0.1 | 1.7 ± 0.5 | 1.4 ± 0.3 | 1.8 ± 0.5 | **<0.001** | **<0.001** |
| LVEDD, mm | 47.8 ± 1.8 | 44.5 ± 5.5 | 47.7 ± 6.0 | 43.0 ± 4.5 | **0.001** | **0.001** |
| LVESD, mm | 35.1 ± 1.5 | 27.4 ± 5.7 | 30.6 ± 5.4 | 25.9 ± 5.2 | **<0.001** | **0.001** |
| LVOT PG, mmHg | 3.5 ± 0.9 | 58.5 ± 48.0 | 8.2 ± 5.2 | 82.2 ± 40.1 | **<0.001** | **<0.001** |
| LVEF, % | 66.3 ± 2.6 | 66.9 ± 5.9 | 66.5 ± 4.8 | 67.1 ± 6.4 | 0.531 | 0.789 |
| LVFS, % | 35.5 ± 2.4 | 36.3 ± 6.7 | 35.8 ± 4.8 | 36.6 ± 7.4 | 0.142 | 0.577 |
| SAM, n (%) | 0 (0) | 54 (69.2) | 3 (12.0) | 51 (96.2) | **<0.001** | **<0.001** |

Abbreviations as in Supplemental Table 1.

**Supplemental Table 3. Echocardiography characteristic in WT and mir15a/mir16-1 CKO mice before and after TAC.**

|  | **0W** |  |  | **2W** |  |  | **4W** |  |  | **8W** |  |  |
| --- | --- | --- | --- | --- | --- | --- | --- | --- | --- | --- | --- | --- |
|  | **WT** | **CKO** |  | **WT** | **CKO** |  | **WT** | **CKO** |  | **WT** | **CKO** |  |
| EF (%) | 70.6±3.5 | 70.1±7.1 |  | 58.7±5.4 | 57.5±9.4 |  | 57.9±5.8 | 47.8±4.6* |  | 42.4±4.6 | 34.5±3.3* |  |
| FS (%) | 37.2±4.4 | 38.9±5.6 |  | 30.4±3.9 | 29.9±6.2 |  | 29.2±4.8 | 23.2±2.1* |  | 21.5±3.9 | 17.2±2.3* |  |
| IVS;d (mm) | 0.90±0.20 | 0.92±0.12 |  | 0.89±0.07 | 1.16±0.27 |  | 1.12±0.28 | 1.16±0.27 |  | 1.09±0.16 | 1.56±0.24* |  |
| IVS;s (mm) | 1.34±0.24 | 1.41±0.26 |  | 1.38±0.18 | 1.50±0.33 |  | 1.45±0.26 | 1.50±0.33 |  | 1.38±0.21 | 1.65±0.13* |  |
| LVID;d (mm) | 3.49±0.12 | 3.38±0.41 |  | 3.42±0.30 | 3.68±0.54 |  | 3.46±0.37 | 3.68±0.54 |  | 3.81±0.15 | 4.04±0.25 |  |
| LVID;s (mm) | 2.26±0.27 | 2.08±0.39 |  | 2.38±0.12 | 2.60±0.57 |  | 2.44±0.26 | 2.60±0.57 |  | 2.92±0.22 | 3.29±0.32* |  |
| LVPW;d (mm) | 0.93±0.14 | 0.75±0.12 |  | 1.00±0.13 | 0.86±0.17 |  | 1.23±0.24 | 0.86±0.17 |  | 0.91±0.15 | 1.07±0.08* |  |
| LVPW;s (mm) | 1.22±0.20 | 1.03±0.19 |  | 1.23±0.24 | 1.04±0.28 |  | 1.23±0.24 | 1.04±0.28 |  | 1.08±0.09 | 1.09±0.11 |  |

*p<0.05 vs. WT (n=8 per group).

Statistical significance was determined by the two-sided t-test.

**Supplemental Table 4. Echocardiography characteristic in WT and mir15a/mir16-1 CKO mice with aging.**

|  | **18W** |  | **24W** |  | **30W** |  | **36W** |  | **42W** |  |
| --- | --- | --- | --- | --- | --- | --- | --- | --- | --- | --- |
|  | **WT(n=12)** | **CKO(n=12)** | **WT(n=12)** | **CKO(n=12)** | **WT(n=12)** | **CKO(n=11)** | **WT(n=11)** | **CKO(n=10)** | **WT(n=11)** | **CKO(n=10)** |
| EF | 76.79±7.08 | 75.26±10.28 | 72.47±7.46 | 71.14±9.12 | 69.86±6.11 | 65.76±6.31 | 66.84±5.39 | 62.62±7.23 | 65.68±7.11 | 55.10±9.85* |
| LV mass (mg) | 76.97±9.17 | 79.91±10.08 | 75.88±11.58 | 74.74±7.13 | 87.02±9.51 | 94.17±9.97 | 90.65±9.94 | 103.41±11.54* | 87.81±11.53 | 97.87±10.54 |
| IVS;d (mm) | 0.86±0.08 | 0.89±0.11 | 0.91±0.11 | 0.90±0.11 | 1.00±0.17 | 0.99±0.13 | 0.99±0.11 | 1.08±0.12 | 0.97±0.11 | 0.97±0.16 |
| IVS;s (mm) | 1.32±0.19 | 1.26±0.19 | 1.35±0.09 | 1.37±0.19 | 1.41±0.09 | 1.52±0.12* | 1.42±0.12 | 1.53±0.10* | 1.43±0.12 | 1.46±0.15 |
| LVID;d (mm) | 3.35±0.40 | 3.27±0.35 | 3.36±0.19 | 3.35±0.22 | 3.48±0.24 | 3.54±0.36 | 3.29±0.23 | 3.48±0.24 | 3.22±0.44 | 3.53±0.49 |
| LVID;s (mm) | 2.02±0.29 | 2.03±0.31 | 2.30±0.29 | 2.26±0.28 | 2.08±0.33 | 2.24±0.33 | 1.93±0.31 | 2.31±0.31* | 2.01±0.31 | 2.44±0.50* |
| LVPW;d (mm) | 0.90±0.10 | 0.93±0.15 | 0.88±0.18 | 0.86±0.11 | 1.02±0.16 | 0.98±0.22 | 0.83±0.11 | 0.89±0.10 | 0.95±0.18 | 1.02±0.22 |
| LVPW;s (mm) | 1.26±0.21 | 1.29±0.18 | 1.24±0.19 | 1.24±0.11 | 1.21±0.14 | 1.27±0.30 | 1.13±0.11 | 1.22±0.16 | 1.16±0.14 | 1.20±0.20 |

*p<0.05 vs. WT. Statistical significance was determined by the two-sided t-test.

**Supplemental Table 5. Echocardiography characteristic in mice received CHO-PEGA-mirNC or CHO-PEGA-mir15a/mir16-1 treatment before and after TAC.**

|  | **0W** |  |  | **2W** |  |  | **4W** |  |  |
| --- | --- | --- | --- | --- | --- | --- | --- | --- | --- |
|  | **mirNC** | **mir15a/mir16-1** |  | **mirNC** | **mir15a/mir16-1** |  | **mirNC** | **mir15a/mir16-1** |  |
| EF (%) | 75.3±7.4 | 76.8±4.8 |  | 64.4±3.7 | 64.9±3.8 |  | 56.7±2.7 | 62.4±3.4* |  |
| FS (%) | 43.3±6.4 | 44.2±4.6 |  | 34.2±2.7 | 34.8±2.9 |  | 29.7±1.1 | 32.5±1.7* |  |
| IVS;d (mm) | 1.04±0.08 | 1.56±0.16 |  | 1.10±0.17 | 1.13±0.17 |  | 1.16±0.14 | 1.31±0.07 |  |
| IVS;s (mm) | 1.64±0.25 | 1.11±0.15 |  | 1.32±0.26 | 1.46±0.16 |  | 1.36±0.12 | 1.60±0.13* |  |
| LVID;d (mm) | 3.24±0.16 | 3.00±0.18 |  | 3.25±0.26 | 3.62±0.24 |  | 3.71±0.14 | 3.23±0.41 |  |
| LVID;s (mm) | 1.85±0.10 | 1.67±0.19 |  | 2.14±0.19 | 2.36±0.17 |  | 2.61±0.12 | 2.18±0.22* |  |
| LVPW;d (mm) | 0.98±0.11 | 0.97±0.11 |  | 0.85±0.14 | 0.84±0.13 |  | 0.88±0.10 | 1.22±0.25* |  |
| LVPW;s (mm) | 1.39±0.23 | 1.34±0.16 |  | 0.95±0.10 | 1.09±0.19 |  | 1.18±0.13 | 1.59±0.23* |  |

*p<0.05 vs. CHO-PEGA-mirNC (n=6 per group).

Statistical significance was determined by the two-sided t-test.

**Supplemental Table 6. Clinical characteristics of hypertensive patients according to LVH.**

|  | **LVH (n=32)** | **Non-LVH(n=228)** | | **P values** |
| --- | --- | --- | --- | --- |
| **Demographics** |  |  | |  |
| Age, y | 50.4±13.0 | 49.4±13.3 | | 0.670 |
| Male, n (%) | 22 (68.8%) | 138 (60.5%) | | 0.371 |
| BMI, kg/m^2^ | 27.0±3.2 | 26.4±4.1 | | 0.215 |
| Obesity, n (%) | 14 (43.8) | 72 (31.6) | | 0.171 |
| SBP at admission, mmHg | 138.0±14.9 | 138.4±17.5 | | 0.902 |
| DBP at admission, mmHg | 86.9±10.5 | 87.5±14.2 | | 0.996 |
| Smoking history, n (%) | 8 (25.0%) | 65 (28.5%) | | 0.679 |
| **Comorbidity** |  |  | |  |
| Diabetes mellitus, n (%) | 4 (12.5%) | 44 (19.3%) | | 0.353 |
| Chronic kidney disease, n (%) | 4 (12.5%) | 17 (7.5%) | | 0.526 |
| Coronary heart disease, n (%) | 6 (18.8%) | 33 (14.5%) | | 0.711 |
| **Medication treatment** |  |  | |  |
| ACEI/ARB, n (%) | 30 (93.8%) | 158 (69.3%) | | **0.004** |
| *β*-Blocker, n (%) | 16 (50.0%) | 111 (48.7%) | | 0.889 |
| Calcium channel blocker, n (%) | 21 (65.6%) | 158 (69.3%) | | 0.674 |
| Diuretics, n (%) | 9 (28.1%) | 37 (16.2%) | | 0.099 |
| **Echocardiography** | |  |  |  |
| IVS, mm | 9.8±1.1 | 9.5±1.1 | | 0.307 |
| LVPW, mm | 9.6±1.1 | 9.2±1.1 | | 0.125 |
| LVEDD, mm | 48.8±5.0 | 47.3±4.7 | | 0.259 |
| LVESD, mm | 31.0±4.6 | 30.1±4.3 | | 0.320 |
| LVMi, g/m^2.7^ | 40.0±8.3 | 39.5±8.5 | | 0.111 |
| LVEF, % | 64.5±4.6 | 65.2±5.4 | | 0.083 |
| **Laboratory data** |  |  | |  |
| Total cholesterol, mmol/L | 4.8±1.3 | 4.8±1.0 | | 0.832 |
| HDL cholesterol, mmol/L | 1.3±0.3 | 1.2±0.3 | | 0.306 |
| LDL cholesterol, mmol/L | 2.8±1.2 | 2.9±0.8 | | 0.477 |
| Creatinine, μmol/L | 71.4±17.2 | 68.4±17.8 | | 0.324 |
| eGFR, ml/min/1.73 m2 | 103.6±24.0 | 106.0±23.1 | | 0.586 |

LVH=left ventricular hypertrophy; LVMi=left ventricular mass index; BMI=body mass index; DBP=diastolic blood pressure; eGFR=estimated glomerular filtration rate; SBP=systolic blood pressure. Other abbreviations as in Supplemental Table 1.
